# Nobiletin Ameliorates Doxorubicin-Induced Nephrotoxicity by Targeting Oxidative Stress, Inflammatory, and Apoptotic Pathways: Combined *In Vivo* and *In Silico* Insights

**DOI:** 10.1021/acsomega.5c07049

**Published:** 2026-01-23

**Authors:** Sümeyra Çetinkaya, İpek Süntar, Mürşide Ayşe Demirel, İlknur Çınar Ayan, Özen Akarca Dizakar

**Affiliations:** 1 Biotechnology Research Center, 655388 Field Crops Central Research Institute, Ankara 06170, Türkiye; 2 Department of Pharmacognosy, Faculty of Pharmacy, 37511 Gazi University, Ankara 06630, Türkiye; 3 Department of Basic Pharmaceutical Sciences, Faculty of Pharmacy, 37511 Gazi University, Ankara 06630, Türkiye; 4 Department of Medical Biology, Faculty of Medicine, Necmettin Erbakan University, Konya 42090, Türkiye; 5 Department of Histology and Embryology, Faculty of Medicine, Bakırçay University, İzmir 35665, Türkiye

## Abstract

Doxorubicin (DOX), although effective as a chemotherapeutic agent, induces significant nephrotoxicity, limiting its clinical application. This study investigated the nephroprotective potential of nobiletin and tangeretin, in comparison with the reference compound silymarin, against DOX-induced acute kidney injury (AKI) in rats. DOX administration significantly increased the kidney-to-body weight ratio (*p* = 0.0053), elevated serum malondialdehyde (MDA) levels (11.49 ± 1.77 nmol/mL), and decreased serum albumin (ALB) levels (10.23 ± 1.84 mg/mL). These changes were accompanied by upregulation of oxidative (*NRF2* and *HO-1*), apoptotic (*CASP3* and *PARP1*), and inflammatory (*TNF-*α, *IL-1*β, *IL-6*, and *NF-*κ*B*) gene and protein markers. Treatment with flavonoids significantly reversed these effects. Improvements in ALB, MDA, and histopathological scores were observed in all treatment groups without significant intertreatment differences. Nobiletin notably reduced MDA to 8.12 ± 1.56 nmol/mL and restored ALB to 15.89 ± 1.31 mg/mL (*p* < 0.01) while also downregulating *CASP3*, *TNF-*α, and *PARP1* expressions. Histopathological analysis showed that nobiletin provided substantial protection against tubular necrosis, glomerular shrinkage, and interstitial inflammation, achieving damage scores similar to the sham group. Furthermore, nobiletin and silymarin both significantly reduced fibrotic area, whereas tangeretin showed moderate effects. Molecular docking revealed that silymarin had the most favorable binding affinities to targets such as CASP3, TNF-α, and HO-1; however, its poor pharmacokinetic properties (TPSA = 155.14 Å^2^) limited its systemic efficacy. In contrast, nobiletin exhibited superior oral absorption (TPSA < 90 Å^2^) and dual CYP inhibition, contributing to better *in vivo* performance. The NRF2/HO-1 pathway was moderately activated, with HO-1 protein levels elevated despite reduced mRNA, suggesting post-transcriptional regulation. In conclusion, nobiletin displayed a consistent multilevel protective profile, with notable modulation of key apoptotic and inflammatory markers, while all compounds provided comparable biochemical and histological benefits. These findings support further development of nobiletin as a promising nephroprotective agent in chemotherapeutic settings.

## Graphical abstract

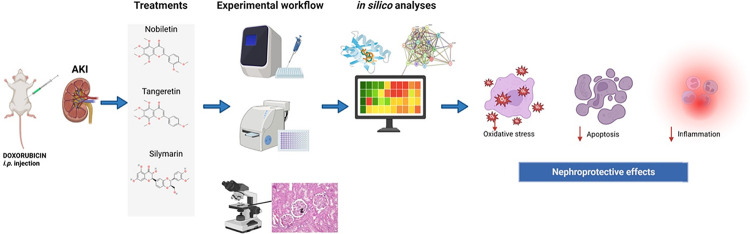

## 1 Introduction

Acute kidney injury (AKI) is clinically defined by an impairment in renal function, leading to the accumulation of nitrogenous waste products and fluid-electrolyte imbalance.^1^ Among the numerous causes of AKI, drug-induced nephrotoxicity remains a critical concern, particularly in oncology settings. Doxorubicin (DOX), a widely used anthracycline chemotherapeutic agent, has demonstrated efficacy against various malignancies, including breast, gastric, hepatocellular carcinoma, endometrial, osteosarcoma, and liver cancers.^2–7^ Its antineoplastic effect primarily stems from its ability to intercalate into DNA and inhibit topoisomerase II, thereby obstructing replication and transcription. Beyond its direct DNA intercalating effects, DOX promotes excessive ROS generation in renal mitochondria, leading to lipid peroxidation, protein oxidation, and DNA damage. This redox imbalance activates pro-inflammatory cascades–most notably NF-κB signaling–which upregulates cytokines such as TNF-α, IL-1β, and IL-6, further amplifying tubular injury. Persistent oxidative stress and inflammation contribute to endothelial dysfunction, disruption of glomerular filtration barrier integrity, and stimulation of profibrotic mediators, ultimately resulting in interstitial fibrosis and chronic kidney damage.^8^ In addition, DOX disrupts mitochondrial electron transport, generating excessive reactive oxygen species (ROS) and triggering oxidative stress, mitochondrial dysfunction, and apoptosis.^9^ Recent evidence indicates that, beyond oxidative stress and apoptosis, DOX can also trigger alternative regulated cell death pathways, including ferroptosis and pyroptosis, thereby amplifying renal tissue injury through lipid peroxidation, iron overload, and inflammasome activation.^10^

Despite its widespread application, DOX’s clinical use is limited by dose-dependent toxicities and the emergence of chemoresistance.^11^ One of the most significant adverse effects is nephrotoxicity, which tends to appear in a delayed fashion and may persist long after treatment. This phenomenon is attributed to DOX’s rapid hepatic metabolism coupled with its prolonged retention in renal tissues.^12,13^ Histologically, DOX-associated kidney injury is characterized by tubular dilation, glomerular atrophy, interstitial fibrosis, and podocyte injury, reflecting severe structural and functional impairments.^14^ These limitations have prompted increasing interest in identifying natural compounds with nephroprotective potential and minimal systemic side effects.

Flavonoids derived from *Citrus* species have gained attention for their wide range of biological properties, including antioxidant, anti-inflammatory, antiapoptotic, and cytoprotective activities. Several studies have highlighted the role of these phytochemicals in mitigating tissue injury induced by chemotherapeutic agents.^15–19^ Among them, nobiletin, a polymethoxylated flavone possessing six methoxy groups, has shown protective effects in cardiovascular, hepatic, and renal models.^20^ It has also been suggested to enhance DOX efficacy and modulate multidrug resistance mechanisms.^21,22^ Tangeretin, structurally similar but with five methoxy groups, has also demonstrated protective effects in nephrotoxicity models.^23,24^

However, despite their structural similarity, limited *in vivo* studies have systematically compared the nephroprotective capacities of nobiletin and tangeretin in the context of DOX-induced AKI. More importantly, a comprehensive investigation into their structure–activity relationships (SAR) and associated molecular mechanisms–including antioxidant defense, inflammatory modulation, and apoptosis regulation–remains lacking. Addressing this gap, the current study evaluates the protective potential of nobiletin and tangeretin in a DOX-induced AKI rat model, using a multifaceted approach. In this study, we systematically evaluate their nephroprotective potential alongside silymarin, a well-established natural hepatoprotective and nephroprotective reference compound widely used in experimental toxicology due to its multitarget antioxidant and anti-inflammatory actions.^25–27^ A combination of biochemical, molecular, histopathological, and computational analyses was employed to clarify their mechanisms of action and potential therapeutic relevance.

## 2 Materials and Methods

### 2.1 Drugs and Chemicals

Doxorubicin (ADRIAMYCIN, 2 mg/mL) was purchased from Pfizer, Türkiye. Nobiletin (Cas no 478–01–3), tangeretin (Cas no 481–53–8), and silymarin (Cas no 65666–07–1) were obtained from Sigma-Aldrich (St. Louis, MO, USA) and dissolved in 0.5% carboxymethyl cellulose (CMC) solution for oral administration. All other chemicals used in this study were of analytical grade.

### 2.2 Experimental Animals and Ethical Approval

Thirty adult male Sprague–Dawley rats (8 weeks old, weighing 190 ± 40 g) were obtained from the Gazi University Laboratory Animals Production and Research Center. The animals were housed under standard laboratory conditions (22 ± 2 °C, 12-h light/dark cycle) and fed a standard pellet diet and drinking water *ad libitum*. All experimental procedures were approved by the Gazi University Local Animal Ethics Committee (Approval No: G.Ü.ET-24.020) and carried out in accordance with the European Union Directive 2010/63/EU, the ARRIVE guidelines, and national regulations for the care and use of laboratory animals.

### 2.3 Experimental Design

Acute nephrotoxicity was induced by a single intraperitoneal (*i.p.*) injection of DOX (15 mg/kg) on day 3 of the experiment. Rats were randomly divided into five groups (n = 6 per group):

1. Group 1 (Sham): No treatment.
2. Group 2 (Control): Received 0.5% CMC orally for 14 consecutive days and a single *i.p.* injection of DOX (15 mg/kg) on day 3.
3. Group 3 (Nobiletin): Received nobiletin (20 mg/kg/day, *p.o.,* in 0.5% CMC) for 14 days, with DOX administered *i.p.* on day 3.
4. Group 4 (Tangeretin): Received tangeretin (20 mg/kg/day, *p.o.,* in 0.5% CMC) for 14 days, with DOX administered *i.p.* on day 3.
5. Group 5 (Reference): Received silymarin (20 mg/kg/day, *p.o.*, in 0.5% CMC) for 14 days, with DOX administered *i.p.* on day 3.

Silymarin was selected as the reference compound because of its well-documented antioxidant, anti-inflammatory, and nephroprotective properties in both preclinical and clinical settings, and its frequent use as a positive control in experimental nephrotoxicity models.^25–27^ Doses and application protocols were selected based on previous studies.^28,29^ On the final day, body weights were recorded prior to anesthesia with ketamine/xylazine (80:10 mg/kg, *i.p*) for sample collection procedures, and the experiment was subsequently terminated.

### 2.4 Sample Collection and Processing

Final body weights and kidney weights of rats in the sham, control, nobiletin, tangeretin, and reference groups were recorded. Following anesthesia, blood samples were collected via cardiac puncture into heparinized tubes. Samples were centrifuged at 1000 rpm for 10 min at room temperature, and the serum was separated and stored at – 20 °C for biochemical analyses. The left kidney was excised and divided into two portions.^30^ One half was homogenized in 1:9 (w/v) ice-cold 50 mM phosphate-buffered saline (PBS, pH 7.4) using a high-speed homogenizer (Qiagen, Hilden, Germany). The homogenates were centrifuged at 14,000 g for 10 min at 4 °C (Thermo Scientific Sorvall ST 16R, USA), and supernatants were stored at −80 °C for protein analysis. The other half of the same kidney was immediately frozen in liquid nitrogen and stored at −80 °C for molecular analyses. The right kidney was fixed in 10% neutral-buffered formalin for histopathological examination.^30,31^

### 2.5 Serum Biochemical Analysis

Serum malondialdehyde (MDA) and albumin (ALB) levels served as biochemical indicators of oxidative injury and renal function. ALB (Cat. No: 201–11–1235) and MDA (Cat. No: 201–11–0157) were measured using ELISA kits (Sunred Biological Technology, Shanghai, China) according to the manufacturer’s instructions. The absorbance was measured at 450 nm using a microplate reader (Thermo Multiskan GO), and results were expressed as mg/mL for ALB and nmol/mL for MDA.

### 2.6 Molecular Analysis

#### 2.6.1 Gene Expression Profiling (RT-qPCR)

To investigate the modulatory effects of nobiletin and tangeretin on gene expression associated with oxidative stress, apoptosis, and inflammation in DOX-induced kidney injury, the transcriptional levels of selected target genes were quantified by RT-qPCR analysis. Total RNA was isolated from frozen renal tissue samples using TRIzol Reagent (Thermo Fisher Scientific, USA) according to the manufacturer’s protocol. The quality and quantity of RNA were assessed by spectrophotometry, and cDNA was synthesized using the iScript cDNA Synthesis Kit (Bio-Rad, Cat. No. 170–8891). Expression levels of genes involved in apoptosis (*BAX, BCL-2, CASP3, CASP8, CASP9, CYCS, PARP1, TP53*), inflammation (*TNF-*α*, IL-1*β*, IL-6, NF-*κ*B p65, ERK1/2, AP-1, p38 MAPK*), and oxidative stress pathways (*NRF2, HO-1, Keap1*) were evaluated using RT-qPCR. *ACTB* and *CYPA* were used as internal reference genes. Gene selection was based on a dual strategy integrating literature review, such as PubMed (https://pubmed.ncbi.nlm.nih.gov), and molecular docking results. Genes relevant to nephrotoxicity were prioritized based on their high binding affinity to compounds and their involvement in key cellular pathways. RT-qPCR amplification was performed using BrightGreen 2X qPCR MasterMix (Applied Biological Materials Inc., Canada) on a Bio-Rad CFX Connect Real-Time PCR System. Primer sequences for target and reference genes were designed using IDT PrimerQuest Tool (https://eu.idtdna.com/Primerquest/Home/Index). Relative expression levels were calculated using the 2^−ΔΔCt method. All reactions were run in triplicate and included no-template controls to assess specificity.

#### 2.6.2 Protein Expression (ELISA)

The protein expression levels of TNF-α, HO-1, and CASP3 in renal tissue homogenates were quantified using commercially available rat-specific ELISA kits (TNF-α: Cat. No. 201–11–0765; HO-1: Cat. No. 201–11–0677; Caspase-3: Cat. No. 201–11–0281; Sunred Biological Technology, Shanghai, China). Assays were performed according to the manufacturer’s instructions. Absorbance was measured at 450 nm using a microplate reader (Thermo Multiskan GO). The concentrations of each protein were normalized to total protein content determined by the Bradford method and expressed as ng/L protein for TNF-α and ng/mL protein for HO-1 and caspase-3.

### 2.7 Histopathological Analysis

To confirm tissue damage at the structural level caused by DOX and to evaluate morphological alterations in the kidney (glomerular and tubular injury, fibrosis, and inflammation), histopathological analyses were performed. Kidney tissues were processed through routine histological procedures, and 4 μm-thick sections were obtained from paraffin-embedded blocks. For the assessment of renal tubular damage, sections were stained with hematoxylin and eosin (H&E), and for the evaluation of fibrosis, Masson’s Trichrome staining was performed. Tubular injury was scored semiquantitatively under light microscopy based on the extent of tubular necrosis, brush border loss, cast formation, tubular dilatation, and degeneration, using the following scale: 0 (normal), 1 (<25%), 2 (25–50%), 3 (50–75%), and 4 (>75% affected area).^32^ Fibrotic changes were quantified by calculating the percentage of fibrotic areas in Masson-stained sections using ImageJ software (NIH, USA), as previously described.^33^ All evaluations were performed using a ZEISS Axioscope 5 computer-assisted light microscope system.

### 2.8 *In* *Silico* Analyses

#### 2.8.1 Pharmacokinetic and Drug-Likeness Evaluation

The pharmacokinetic properties and drug-likeness profiles of nobiletin and tangeretin were evaluated using the SwissADME web tool (http://www.swissadme.ch/). Canonical SMILES strings were retrieved from the PubChem database and used as input: nobiletin (SMILES: COC1 = C­(C = C­(C = C1)­C2 = CC­(=O)­C3 = C­(O2)­C­(=C­(C­(=C3OC)­OC)­OC)­OC)­OC), tangeretin (SMILES: COC1 = CC = C­(C = C1)­C2 = CC­(=O)­C3 = C­(O2)­C­(=C­(C­(=C3OC)­OC)­OC)­OC), and silymarin (SMILES: COC1 = C­(C = CC­(=C1)­C2C­(OC3 = C­(O2)C = C­(C = C3)­C4C­(C­(=rO)­C5 = C­(C = C­(C = C5O4)­O)­O)­O)­CO)­O). These molecules have respective molecular weights of 402.39 g/mol (nobiletin), 372.36 g/mol (tangeretin), and 482.44 g/mol (silymarin). Canonical SMILES used to calculate gastrointestinal (GI) absorption, blood–brain barrier (BBB) permeability, lipophilicity (LogP), topological polar surface area (TPSA), aqueous solubility, P-glycoprotein (P-gp) substrate likelihood, and cytochrome P450 (CYP) isoform inhibition potential (*CYP1A2, CYP2C19, CYP2C9, CYP2D6*, and *CYP3A4*). The BOILED-Egg model was utilized to predict the likelihood of GI absorption and BBB penetration based on WLOGP and TPSA values. Drug-likeness was assessed using multiple rule-based filters, including Lipinski, Veber, Egan, Ghose, and Muegge, alongside bioavailability score prediction.

#### 2.8.2 Molecular Docking Simulations

To evaluate the binding affinities of compounds toward key redox-regulating enzymes, molecular docking simulations were performed using the CB-Dock2 server (https://cadd.labshare.cn/cb-dock2/index.php), which integrates cavity detection and AutoDock Vina-based scoring. Protein targets included apoptosis, inflammation and oxidative stress pathways: BAX (PDB ID: 6EB6), BCL-2 (PDB ID: 1G5M), CASP3 (PDB ID: 6CKZ), CASP8 (PDB ID: 6PX9), CASP9 (PDB ID: 5JUY), PARP-1 (PDB ID: 4DQY), p53 (PDB ID: 1TUP), CytC (PDB ID: 3ZCF), Nrf2 (PDB ID: 5WFV), Keap1 (PDB ID: 8 × 34), HO-1 (PDB ID: 1N3U), TNF-a (PDB ID: 1TNF), IL-1β (1L1B), IL-6 (PDB ID: 1ALU), NK–κB p65 (PDB ID: 4G3D), p38 MAPK (PDB ID: 2Y8O), AP-1 (PDB ID: 4HMY). Ligands were uploaded as.mol2 files using the CB-Dock server (https://cadd.labshare.cn/cb-dock2/index.php). Docking grid boxes were automatically generated by the server based on predicted binding cavities, with grid dimensions varying depending on the target protein (typically within the range of 23–35 Å^2^). Cavity center coordinates and contact residues were recorded for each docking run. Docking results were ranked according to Vina binding scores (kcal/mol), and the best-scoring poses were selected for further analysis. Binding interactions were evaluated based on hydrogen bonding, hydrophobic contacts, and proximity to catalytically relevant residues. Representative docking poses and detailed interaction profiles, including predicted binding cavities, center coordinates, and contact residues for each protein–ligand complex, are provided in Supplementary File 1 as 2D ligand–protein interaction diagrams.

#### 2.8.3 Protein–Protein Interaction (PPI) Network Analysis

To explore the broader interactome of targets associated with oxidative stress, apoptosis, and inflammation, a PPI network was constructed using the STRING database v12.0 (https://string-db.org), restricted to *Rattus norvegicus* in accordance with the *in vivo* model. Networks were generated using a medium-to-high confidence score threshold (≥0.7), integrating evidence from experiments, curated databases, text mining, coexpression, and protein homology. Edge thickness reflected interaction confidence.

### 2.9 Statistical Analysis

Data obtained from biochemical and molecular assays are presented as mean ± standard deviation (SD), while histopathological data are expressed as mean ± standard error of the mean (SEM). Statistical analyses were performed using GraphPad Prism version 8.0.1 (GraphPad Software, San Diego, CA, USA). Differences among groups were evaluated using one-way analysis of variance (ANOVA) followed by Tukey’s multiple comparison post hoc test. A p-value less than 0.05 was considered statistically significant for all comparisons.

## 3 Results

### 3.1 Effect of Treatments on Final Body Weight and Relative Kidney Weight

Kidney-to-body weight ratios (g/100 g BW) are presented in Figure 1A and differed significantly among the treatment groups (p = 0.0053). The control group exhibited significantly higher kidney-to-body weight ratios compared to the sham (p = 0.0073), nobiletin (p = 0.0172), and silymarin (p = 0.0146) groups (Table 1).

**Table 1:** Effect of Treatment on Body Weight, Kidney Weight, and Relative Kidney Weight in Rats^a^

Group	Final body weight (g) ± SD	Kidney weight (g) ± SD	Kidney/body weight ratio (g/100 g BW) ± SD
**Sham**	265.3 ± 3.55b^b^	1.26 ± 0.314	0.478 ± 0.135^c^
**Control**	201.7 ± 5.1d^d^	1.38 ± 0.270	0.680 ± 0.120^d^
**Nobiletin**	225.5 ± 5.47^c^	1.12 ± 0.135	0.497 ± 0.060^c^
**Tangeretin**	221.5 ± 12.86^c^	1.17 ± 0.159	0.529 ± 0.070^c^
**Silymarin**	225.2 ± 23.49^c^	1.11 ± 0.13	0.493 ± 0.046^c^

a Data are presented as mean ± SD (*n* = 6).

b Significantly different from all treatment and control groups (*p* < 0.05).

c Significantly different from sham and control groups (*p* < 0.05).

d Significantly higher than all other groups (*p < 0.05).

**Figure 1:**
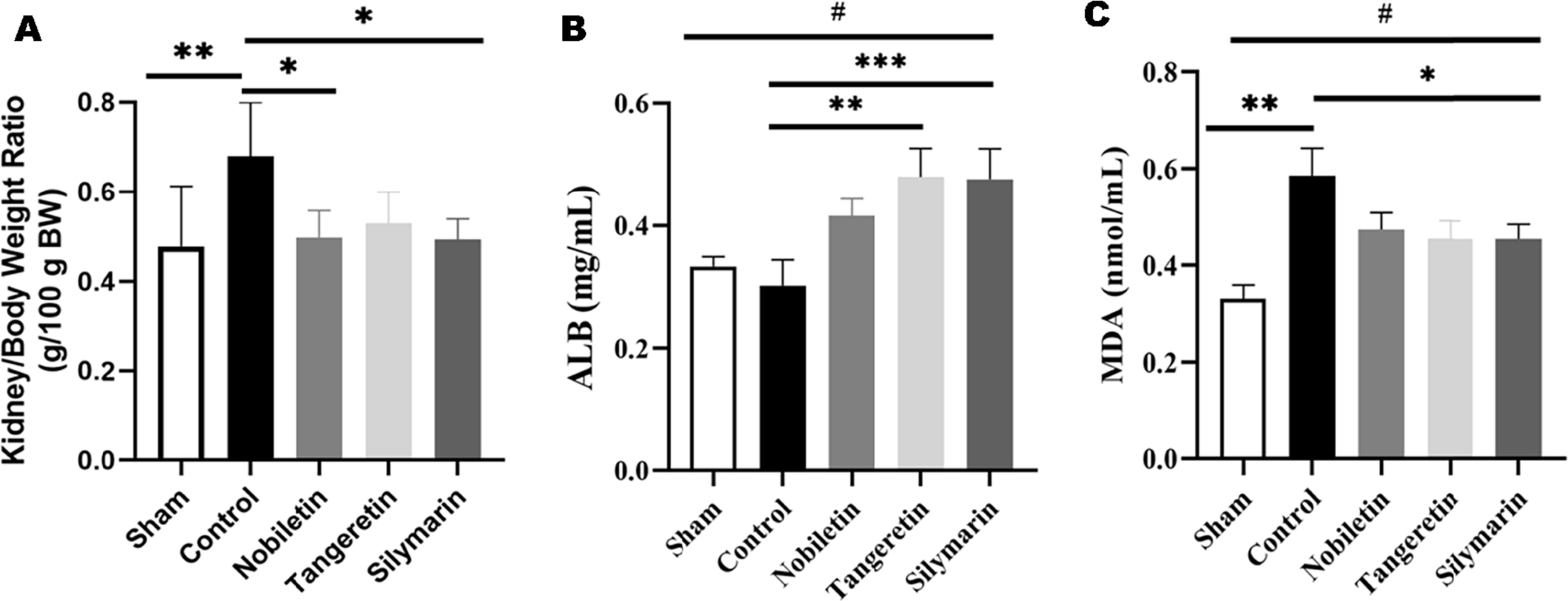
Kidney-to-body weight ratio (g/100 g BW) (A), serum ALB levels (B), and serum MDA levels (C) across experimental groups. Data are presented as mean ± SD (*n* = 6) (*p* < 0.05*, *p* < 0.01**, *p* < 0.001***, *p* < 0.0001^#^).

### 3.2 Biochemical Analyses

Serum albumin (ALB) levels differed significantly among groups (*p* < 0.0001^#^), as shown in Figure 1B. The control group exhibited significantly lower ALB levels (10.23 ± 1.84^a^ mg/mL) compared to the nobiletin (15.89 ± 1.31^b^ mg/mL; *p* < 0.01**), tangeretin (19.22 ± 2.19^c^ mg/mL; *p* < 0.01**), and silymarin (18.83 ± 2.25^c^ mg/mL; *p* < 0.001***) groups. Serum malondialdehyde (MDA) levels significantly increased following DOX administration (*p* < 0.0001^#^) (Figure 1C). The control group showed markedly elevated MDA levels (11.49 ± 1.77^a^ nmol/mL) compared to the sham group (3.41 ± 0.92^c^ nmol/mL; *p* < 0.01**). Nobiletin (8.12 ± 1.56^b^ nmol/mL, *p* < 0.05*), tangeretin (7.22 ± 0.48^b^ nmol/mL, *p* < 0.05*), and silymarin (7.19 ± 0.43^b^ nmol/mL, *p* < 0.05*) treatments significantly reduced MDA levels compared to the control group (Figure 1C). The differences among the treatment groups, however, were not statistically significant for either marker (*p* > 0.05), indicating comparable biochemical improvements across all compounds.

### 3.3 Histopathological Analyses

In the sham group, renal tissue sections displayed normal glomerular and tubular morphology with no observable pathological alterations. In contrast, the control group exhibited severe tubular dilatation, loss of brush border, intracellular vacuolization, and degenerative changes. Marked glomerular atrophy, interstitial inflammation, hyaline accumulation, and edema were also noted. Based on tubular injury scores calculated from 10 randomly selected fields per group, the sham group had the lowest histopathological damage score (0.27 ± 0.06^a^, *p* < 0.05*). The control group exhibited a significantly higher score (3.10 ± 0.09^c^) compared to the nobiletin (2.07 ± 0.08^b^), tangeretin (2.42 ± 0.10^b^), and silymarin (2.33 ± 0.09^b^) groups (*p* < 0.05*). Among the treatment groups, the lowest tubular injury score was observed in the nobiletin group; although this difference was not statistically significant, histological improvements appeared more pronounced (Figure 2). In the analysis of fibrotic area percentage assessed by Masson’s trichrome staining, the sham group showed significantly lower fibrosis levels (0.26 ± 0.03^a^) than the control group (1.49 ± 0.19^c^, *p* < 0.05*). All treatment groups differed significantly from the control group (*p* < 0.05*). Although differences were noted among the nobiletin (0.33 ± 0.05^a^ ^b^), silymarin (0.43 ± 0.06^a^ ^b^), and tangeretin (0.56 ± 0.11^a^ ^b^) groups, these did not reach statistical significance (*p* > 0.05), suggesting similar histological protection by all compounds. Silymarin exhibited lower fibrosis levels compared to tangeretin, though the difference was not statistically significant (Figure 3).

**Figure 2:**
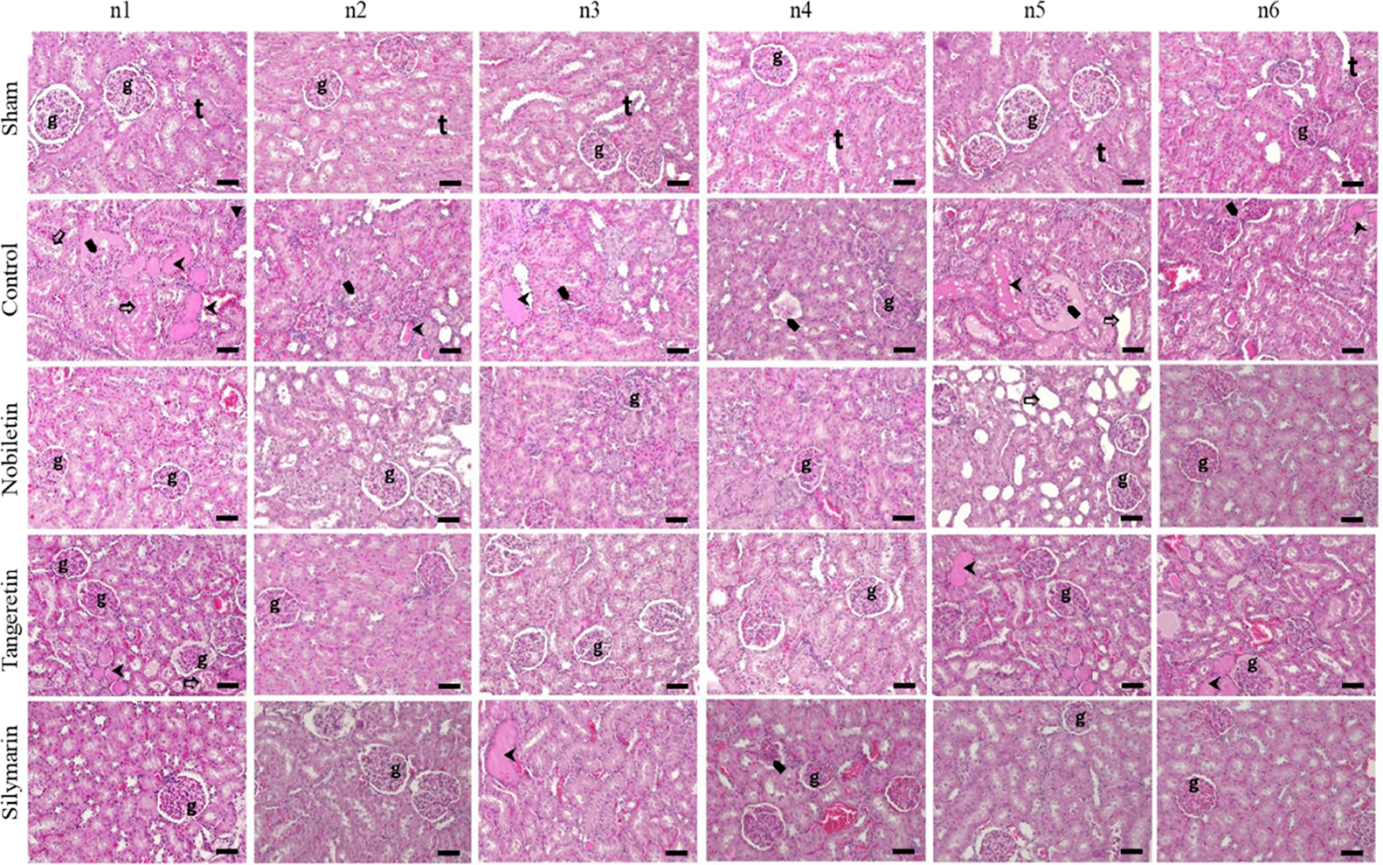
Representative kidney sections from sham, control, nobiletin, tangeretin, and silymarin groups stained with hematoxylin and eosin (H&E, × 200 magnification and scale bar = 50 μm). The sham group displayed normal glomerular (**g**) and tubular (**t**) morphology. Histopathological alterations include glomeruli (**g**), hyaline accumulation (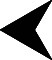), glomerular atrophy (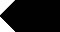), tubular dilatation (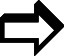), and interstitial inflammation (▼) in the control group. Severe structural damage was observed in the control group, whereas the treatment groups, particularly nobiletin, showed improved renal histology.

**Figure 3:**
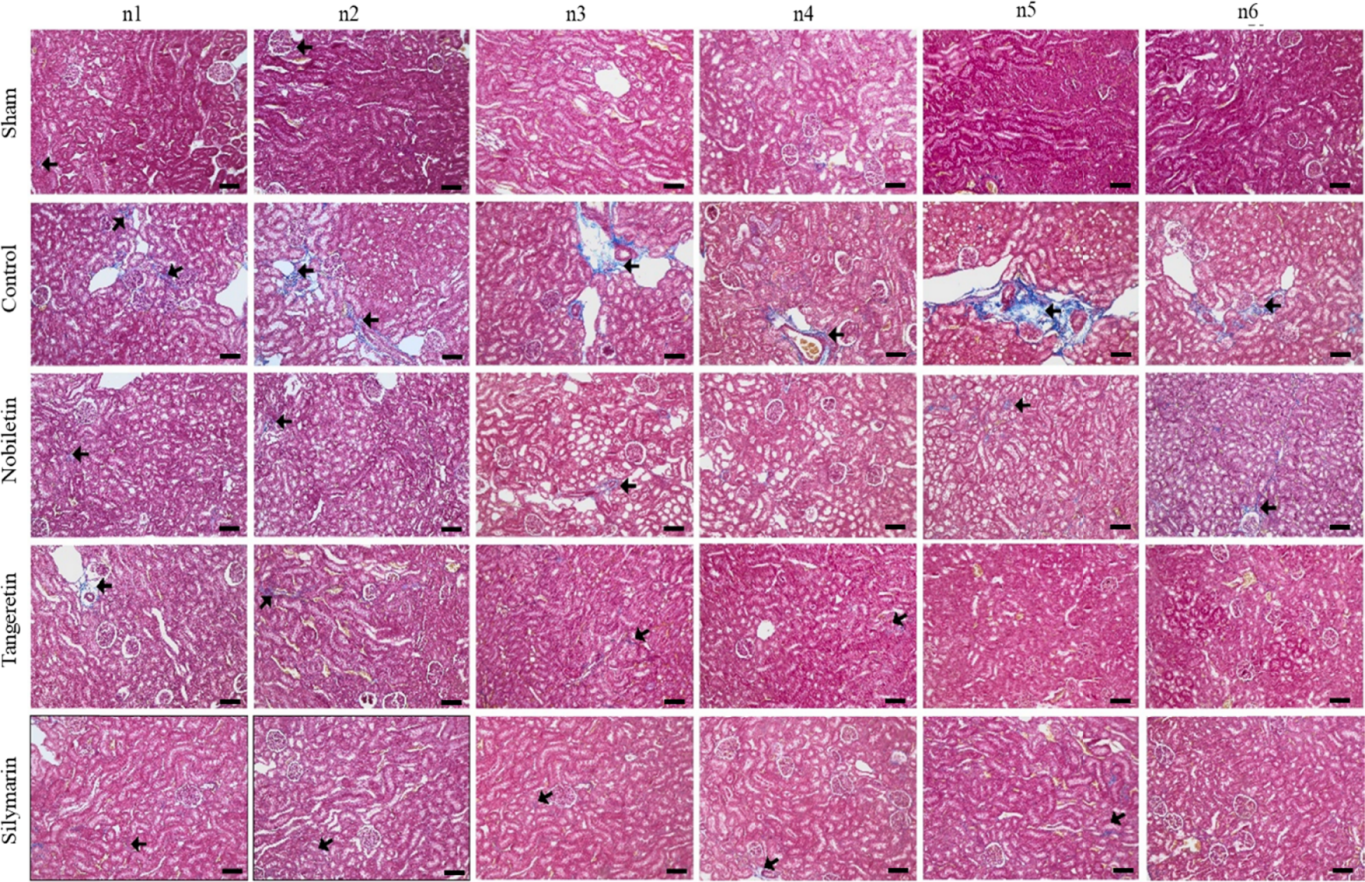
Masson’s trichrome-stained kidney sections from sham, control, nobiletin, tangeretin, and silymarin groups. Blue-stained fibrotic areas (**←**) indicate collagen fiber accumulation. Increased fibrosis is evident in the control group, while reduced fibrotic areas are observed in treatment groups (MT, × 100 magnification and scale bar = 100 μm).

### 3.4 Gene Expression Analyses

#### 3.4.1 Apoptosis-Related Genes

DOX-treatment significantly upregulated the expression of pro-apoptotic genes *BAX*, *CASP3*, *CASP8*, *CASP9*, *CYCS*, *TP53*, and *PARP1*, while downregulating antiapoptotic *BCL-2* expression compared to the sham group (*p* < 0.01 for all). Nobiletin, tangeretin, and silymarin groups showed significant decreases in these pro-apoptotic gene expressions. Specifically, *BAX* expression was downregulated approximately 2.68-fold (p = 0.0008), 2.54-fold (p = 0.0013), and 2.7-fold (p = 0.0006), respectively. *CASP3* levels were also significantly decreased by 2.02-fold (p = 0.0016), 2.3-fold (p = 0.0005), and 3.03-fold (*p* < 0.0001). *CASP8* expression was reduced by 2.1-fold (p = 0.0278), 2.17-fold (p = 0.0465), and 2.6-fold (p = 0.0051), while *CASP9* was suppressed by 4.75-fold (*p* < 0.0001), 3.07-fold (p = 0.0003), and 6.0-fold (*p* < 0.0001), respectively. *PARP1* expression was also highest in the DOX group, with treatment-induced decreases of 2.68-fold (p = 0.017), 2.12-fold (p = 0.057), and 2.0-fold (p = 0.084). *TP53* was significantly downregulated by 4.0-fold (p = 0.0005), 2.4-fold (p = 0.007), and 3.12-fold (p = 0.001) with treatment. *CYCS* expression was significantly decreased by 4.5-fold (p = 0.012), 2.8-fold (p = 0.012), and 3.6-fold (p = 0.015) by the treatments. *BCL-2* expression was increased by 2.05-, 1.62-, and 1.53-fold, but just the dox and sham groups showed statistically significant changes (*p* < 0.01**) (Figure 4).

**Figure 4:**
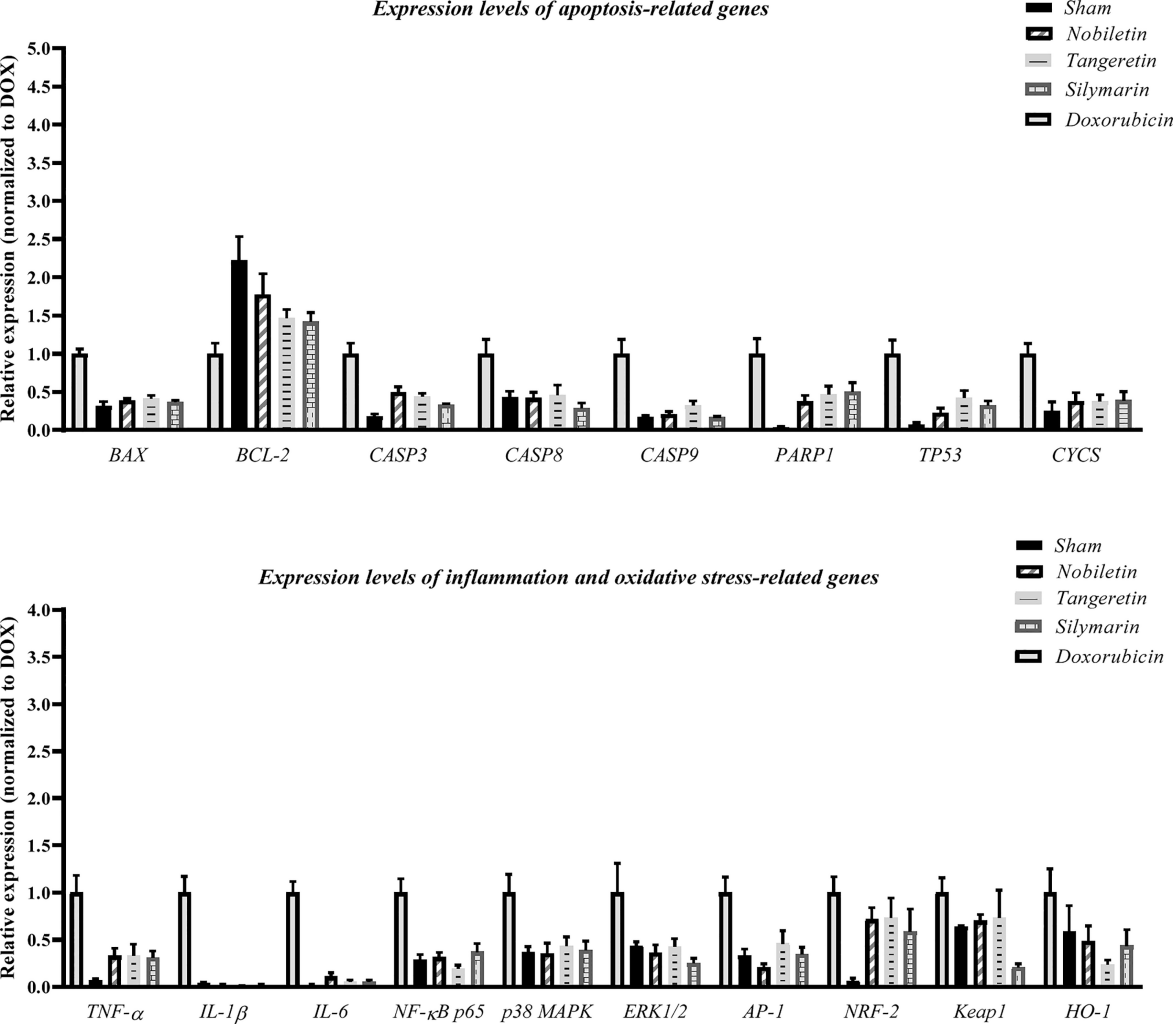
Effects of nobiletin, tangeretin, and silymarin on the expression of apoptosis-related genes, and inflammation- and oxidative stress-related genes in renal tissues. Gene expression levels are presented as fold change relative to the reference group.

#### 3.4.2 Inflammation-Related Genes

DOX treatment led to a significant increase in *TNF-*α, *MAPK3*, *AP-1*, *IL-1*β, *IL-6*, and *NF-*κ*B p65* expressions (*p* < 0.01). *TNF-*α was upregulated in the DOX group and significantly downregulated in the sham (15-fold, *p* < 0.0001), nobiletin and tangeretin (3-fold, *p* < 0.001), and silymarin (2.3-fold, p = 0.0032) groups. IL-1β was reduced by 60-fold, 58-fold, and 55-fold in the nobiletin, tangeretin, and silymarin groups, respectively (*p* < 0.0001). *IL-6* was similarly suppressed by 14-fold, 26-fold, and 19-fold (*p* < 0.0001), while *MAPK3* expression was significantly reduced in the silymarin group (∼3.9-fold, p = 0.022), with nonsignificant trends in nobiletin (∼2.8-fold, p = 0.079) and tangeretin (∼2.4-fold, p = 0.119). AP-1 expression was highest in the DOX group and significantly reduced by 6.5-fold (p = 0.0004) and 4.0-fold (p = 0.0030) in the nobiletin and sham groups, respectively. *NF-*κ*B p65* was reduced by 2.4-fold (sham), 2.9-fold (nobiletin), 4.3-fold (tangeretin), and 3.5-fold (silymarin), with all comparisons reaching statistical significance (*p* < 0.001) (Figure 4).

#### 3.4.3 Oxidative Stress-Related Genes

*NRF2* expression was significantly upregulated in the DOX group, with a 26-fold increase compared to sham (p = 0.0214), indicating oxidative stress. Although the treatment groups showed reduced *NRF2* expression, these changes were not statistically significant. *KEAP1* expression did not show significant differences among groups (p = 0.408), suggesting a *KEAP1*-independent mechanism. *HO-1* expression was significantly upregulated in the DOX group and suppressed by all treatments. *HO-1* was downregulated by 2.1-fold (p = 0.0090), 2.5-fold (p = 0.0031), and 2.9-fold (p = 0.0015) with nobiletin, tangeretin, and silymarin, respectively. *p38 MAPK* expression was significantly elevated in the DOX group compared to sham (2-fold increase, p = 0.0080), and significantly reduced by 1.9-fold (p = 0.0067), 1.6-fold (p = 0.0300), and 1.8-fold (p = 0.0117) with the respective treatments (Figure 4). These results support that nobiletin, tangeretin, and silymarin significantly attenuate DOX-induced pro-apoptotic, inflammatory, and oxidative gene expression changes, contributing to their protective effects.

#### 3.4.4 Protein Expressions

TNF-α protein levels revealed a statistically significant difference among the groups (p = 0.0008). DOX treatment significantly increased TNF-α levels compared to the sham group (p = 0.0167), confirming its pro-inflammatory effect. Among the treatment groups, both nobiletin (p = 0.0058) and silymarin (p = 0.0446) resulted in significantly lower TNF-α levels compared to the DOX group. CASP3 protein concentrations revealed statistically significant differences among the groups (*p* < 0.0001). DOX treatment resulted in a significant increase in CASP3 levels compared to the sham group (p = 0.0006), confirming the apoptotic effect of DOX. Treatment with nobiletin (p = 0.0081), tangeretin (p = 0.0060), and silymarin (p = 0.0196) significantly reduced CASP3 protein levels compared to the control group. These findings indicate that all three compounds exhibited protective effects against DOX-induced caspase-3 activation, with nobiletin showing a numerically grater reduction compared to tangeretin and silymarin. Additionally, nobiletin also showed a significant difference compared to tangeretin (p = 0.0406), suggesting a relatively stronger antiapoptotic effect. For HO-1 protein levels, a significant overall difference was observed among the groups (p = 0.0224). Although DOX treatment increased HO-1 levels compared to sham, this change was not statistically significant (p = 0.2725). In contrast, nobiletin (p = 0.0185), tangeretin (p = 0.0179), and silymarin (p = 0.0202) significantly elevated HO-1 levels relative to the sham group. No significant differences were observed between the control group and any of the treatments (*p* > 0.4), indicating that although phytochemical treatments further promoted HO-1 expression, they did not counteract the HO-1 increase already induced by DOX (Figure 5). This supports a potential synergistic antioxidant response in the treatment groups.

**Figure 5:**
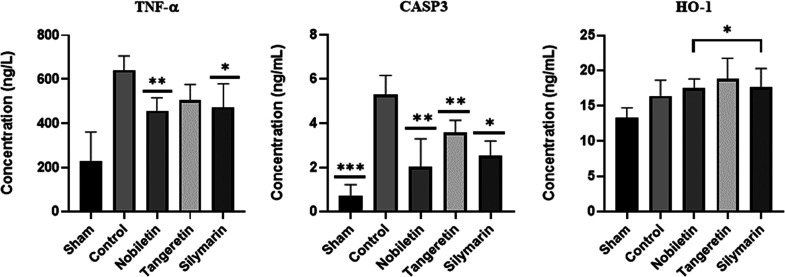
Effects of treatments on TNF-α, CASP3, and HO-1 protein concentrations. TNF-α concentrations were significantly elevated in the DOX (control) group compared to the sham (*p* = 0.0167*). Nobiletin and silymarin treatment significantly reduced TNF-α levels compared to control (*p* = 0.0058**, *p* = 0.044**, respectively), while tangeretin showed no statistically significant decrease vs control (*p* = 0.057). CASP3 levels were significantly increased in the control group vs sham (*p* = 0.0006***). Treatment with nobiletin (*p* = 0.0081**), tangeretin (*p* = 0.0060**), and silymarin (*p* = 0.0196*) significantly reduced CASP3 levels compared to control. HO-1 levels were significantly increased by nobiletin (*p* = 0.0185*), tangeretin (*p* = 0.0179*), and silymarin (*p* = 0.0202*) compared to the sham group. No significant differences were observed when compared to the control group (*p* > 0.4). Values are presented as mean ± SD (*n* = 6). Statistical comparisons were performed using one-way ANOVA followed by Tukey’s posthoc test. *p* < 0.05*, *p* < 0.01**, *p* < 0.001***.

### 3.5 Pharmacokinetics and Drug-Likeness Profiles of Nobiletin, Tangeretin, and Silymarin

Both nobiletin and tangeretin exhibited high predicted GI absorption, suggesting favorable oral bioavailability, whereas silymarin indicated low GI absorption, indicating limited systemic exposure via the oral route. Among the compounds, only tangeretin was predicted to cross the BBB, highlighting its potential for central nervous system (CNS)-related effects. All three compounds were nonsubstrates of P-glycoprotein (P-gp), which may reduce the likelihood of efflux-mediated bioavailability issues. In terms of lipophilicity, nobiletin and tangeretin showed higher iLOGP values (3.96 and 3.71, respectively) than silymarin (2.79), consistent with their improved membrane permeability. With regard to metabolic interactions, nobiletin and tangeretin were predicted to inhibit CYP2C9 and CYP3A4 isoforms, whereas silymarin selectively inhibited CYP3A4. This suggests potential drug–drug interaction risks, particularly when coadministered with substrates of these enzymes. Additionally, aqueous solubility predictions (ESOL) indicated poor solubility for all compounds, though differences were marginal (ranging from – 4.11 to – 4.18). Lastly, topological polar surface area (TPSA) values supported permeability predictions: silymarin had the highest TPSA (155.14 Å^2^), which may limit its passive diffusion, especially across the BBB, whereas nobiletin (85.59 Å^2^) and tangeretin (76.36 Å^2^) were within the favorable range for good absorption and distribution. Collectively, tangeretin showed favorable pharmacokinetic properties, particularly for CNS-targeted applications, while nobiletin also indicates promising systemic bioavailability. Silymarin, despite its therapeutic potential, may require formulation optimization to overcome its limited absorption and permeability characteristics (Table 2).

**Table 2:** Key Pharmacokinetic Properties of the Compounds

Property	Nobiletin	Tangeretin	Silymarin
**GI Absorption**	High	High	Low
**BBB Permeant**	No	Yes	No
**P-gp Substrate**	No	No	No
**CYP Inhibition**	CYP2C9, CYP3A4	CYP2C9, CYP3A4	CYP3A4
**iLOGP**	3.96	3.71	2.79
**ESOL (Solubility)**	–4.18	–4.11	–4.14
**TPSA (Å** **^2^** **)**	85.59	76.36	155.14

The BOILED-Egg analysis revealed distinct absorption and distribution profiles among the tested compounds. Tangeretin was located within both the gastrointestinal absorption (HIA) and blood–brain barrier (BBB) permeation zones, suggesting optimal pharmacokinetic behavior for both systemic and CNS delivery. Nobiletin fell within the HIA region but outside the BBB ellipse, indicating favorable oral absorption yet limited CNS penetration. In contrast, silymarin was positioned outside both HIA and BBB zones, reflecting suboptimal absorption and poor brain accessibility. These findings support tangeretin and nobiletin as pharmacokinetically advantageous candidates, whereas silymarin may require formulation strategies to enhance its bioavailability (Figure 6).

**Figure 6:**
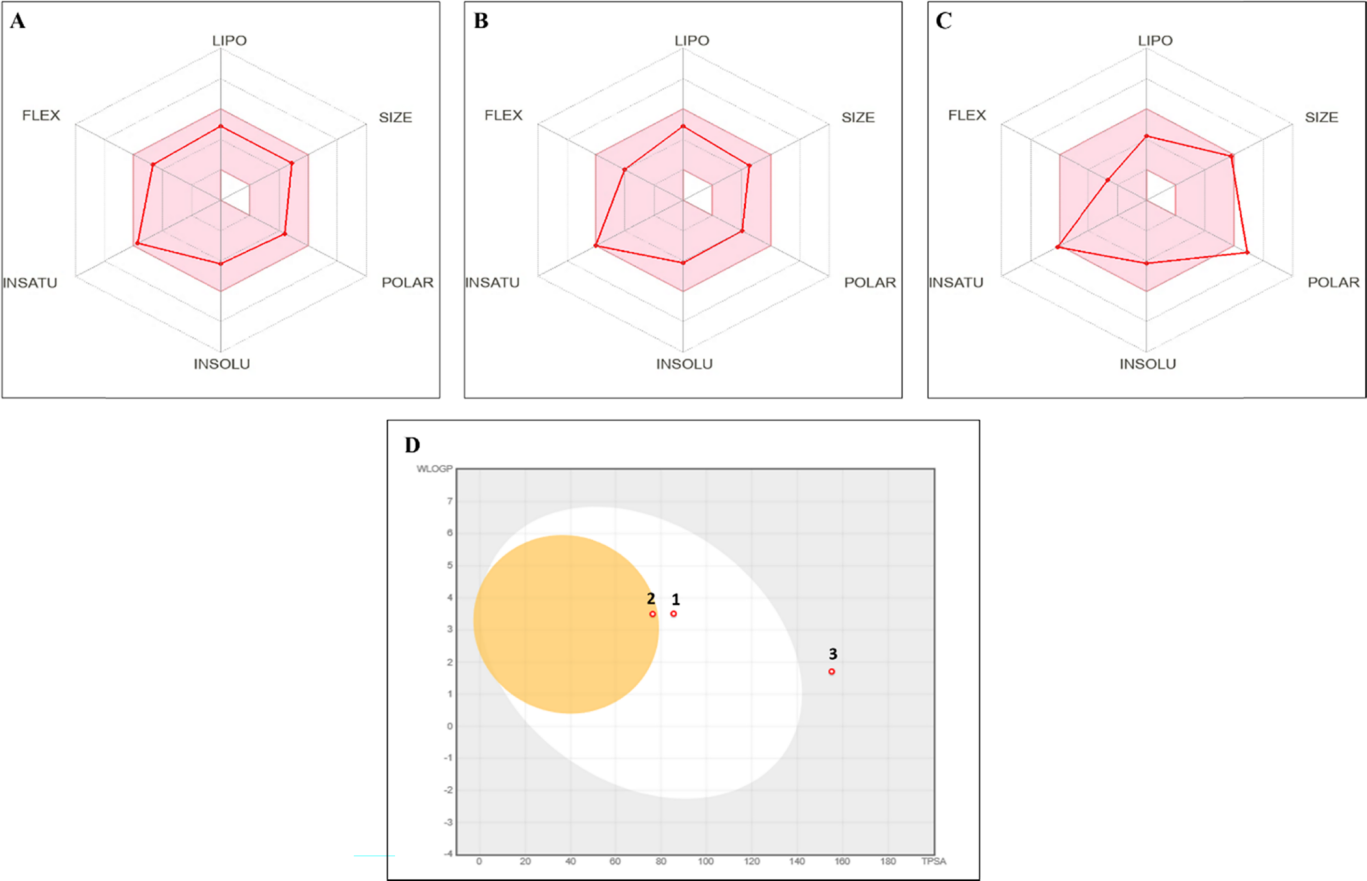
Pharmacokinetic and drug-likeness profiles of nobiletin (A), tangeretin (B), and silymarin (C) predicted by the SwissADME radar model. Each plot displays six key properties: lipophilicity (LIPO), size, polarity (POLAR), insolubility (INSOLU), unsaturation (INSATU), and molecular flexibility (FLEX). These parameters are used to evaluate drug-likeness and oral bioavailability profiles of the compounds. (D) BOILED-Egg plot showing gastrointestinal absorption (GI) (white region) and blood–brain barrier (BBB) permeability (yellow region). The plot illustrates the WLOGP versus TPSA values of the three compounds. Tangeretin falls within both high oral bioavailability (HIA) and BBB regions, suggesting high oral absorption and potential CNS permeability (2). Nobiletin is localized within the HIA zone but outside the BBB ellipse (1). Silymarin, with its high TPSA, is positioned outside both ellipses, reflecting poor predicted GI absorption and BBB impermeability (3).

### 3.6 Molecular Docking

Molecular docking analyses supported that all three compounds–nobiletin, tangeretin, and silymarin–exhibited notable binding affinities toward multiple molecular targets associated with inflammation (TNF-α, IL-1β, IL-6), oxidative stress (NRF2, KEAP1, HO-1), and apoptosis (CASP3, CASP8, CASP9, BAX, TP53). Among them, silymarin generally showed the strongest binding energies (e.g., – 11.7 kcal/mol for AP-1, – 11.1 kcal/mol for CASP8), while nobiletin and tangeretin also displayed favorable interactions, particularly with antioxidant and inflammatory targets. Despite silymarin’s superior docking scores in select targets, the comparable affinities of nobiletin and tangeretin, in combination with their favorable pharmacokinetic profiles, suggest these compounds may represent promising therapeutic alternatives in targeting multiple pathophysiological pathways implicated in DOX-induced kidney injury (Figure 7) (Supplementary File).

**Figure 7:**
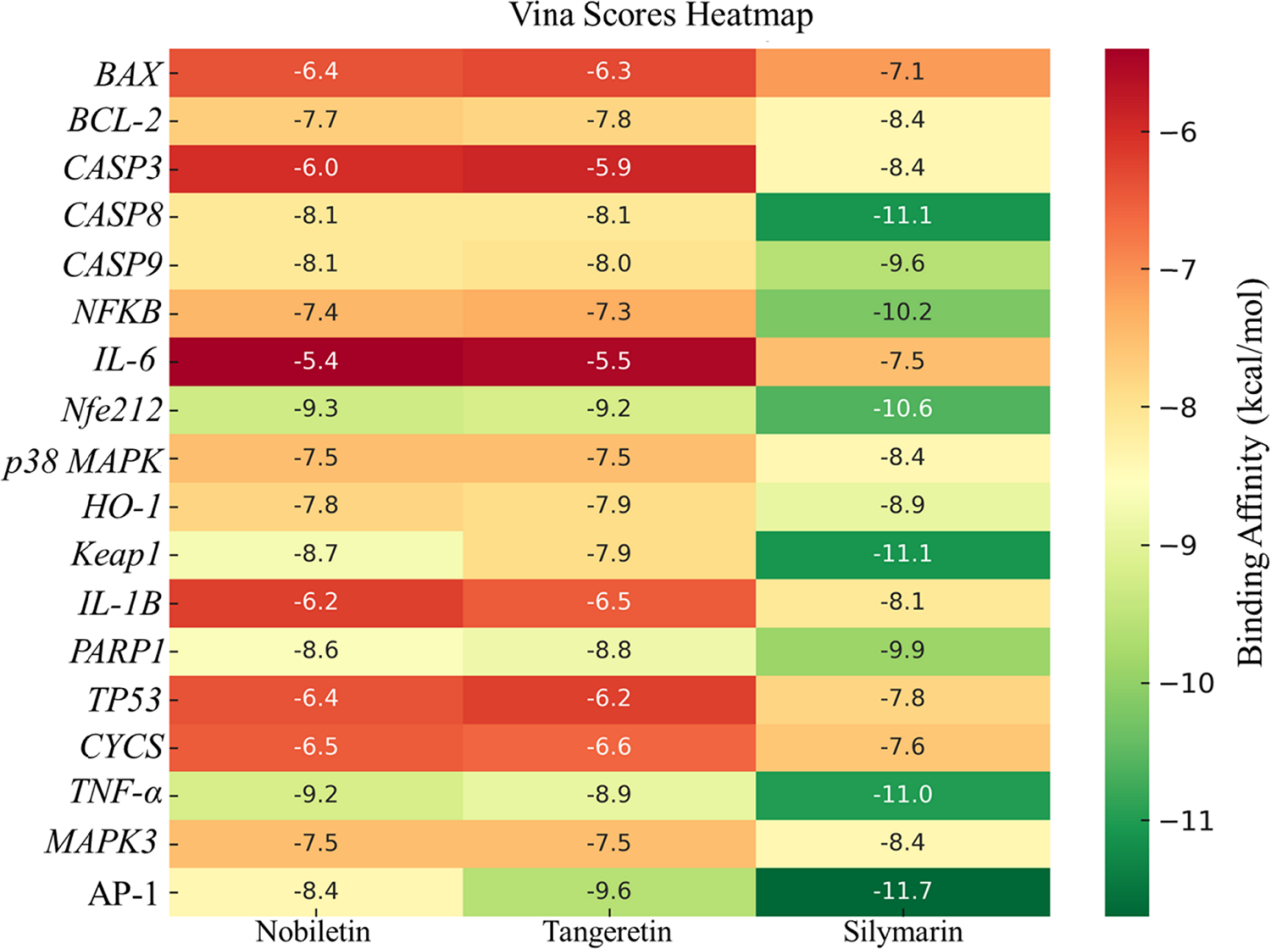
Molecular docking heatmap of vina binding affinities for selected target proteins. The binding affinities (expressed in kcal/mol) of nobiletin, tangeretin, and silymarin were calculated for each protein target using AutoDock Vina. Darker green shades indicate stronger predicted binding affinities (more negative vina scores), whereas red tones reflect weaker interactions. Among the tested compounds, silymarin generally indicated the strongest binding across several targets such as CASP8, AP-1, and KEAP1. However, nobiletin and tangeretin also exhibited favorable binding profiles, particularly for NRF2, NF-κB, and TP53, highlighting their therapeutic potential in DOX-induced nephrotoxicity.

### 3.7 Protein–Protein Interaction (PPI) Network Analysis

To elucidate the molecular interactions among the targets associated with oxidative stress, inflammation, and apoptosis, a PPI network was constructed using the STRING database (*Rattus norvegicus*). The resulting network comprised 14 nodes and 87 edges with a highly significant enrichment (p-value <1.0e–16), indicating nonrandom functional connectivity among apoptosis, inflammation, and oxidative-stress regulators. Central hub proteins such as BAX, CASP3, MAPK3 (representing ERK1/2 and p38 MAPK), LOC103694380 (membrane-bound TNF-α form), IL6, and TP53 exhibited high connectivity, highlighting their pivotal roles in maintaining network stability (Figure 8). Consistent with the *Rattus norvegicus* data set, IL1β and NF-κB p65 were not present in this network.

**Figure 8:**
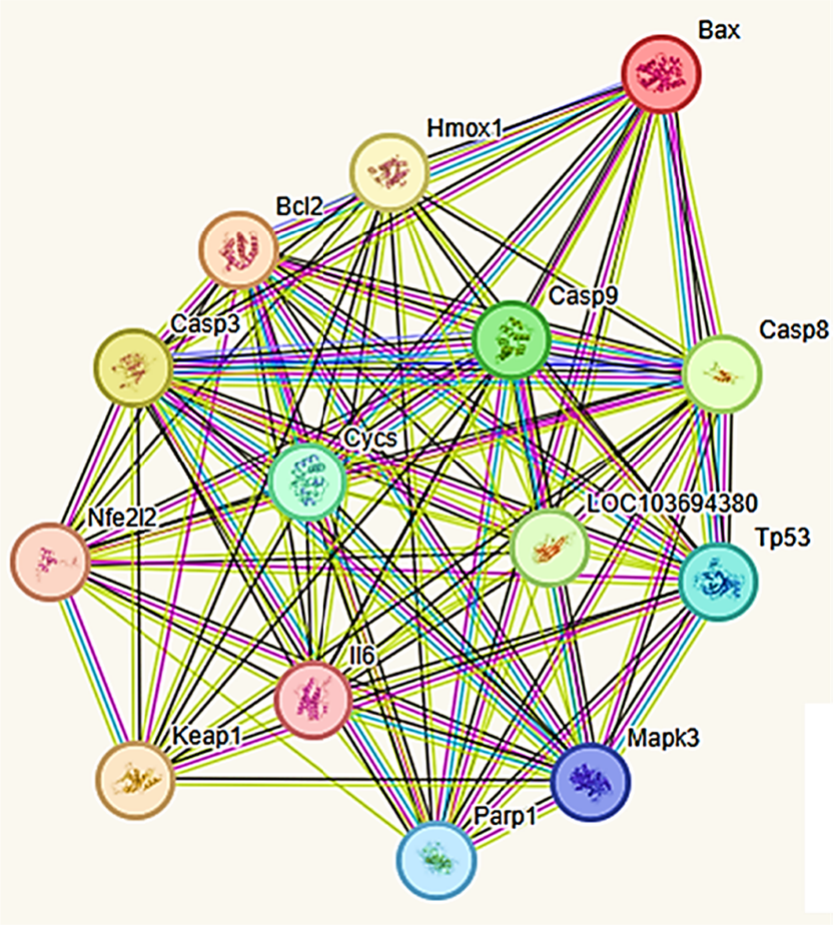
Protein–protein interaction (PPI) network of selected targets constructed using the STRING database (*Rattus norvegicus*). The network comprises 14 nodes and 87 edges, with a significant PPI enrichment p-value (<1.0e–16), indicating a nonrandom distribution of functional interactions. Key apoptotic proteins (CASP3, CASP8, CASP9, BAX, BCL2, PARP1, TP53, CYCS), inflammatory mediators (IL-6, LOC103694380 representing the membrane-bound TNF-α form), and oxidative stress-related regulators (HMOX1, KEAP1, NFE2L2) demonstrate dense interconnectivity, with MAPK3 subsuming ERK1/2 and p38 MAPK functions. Edge colors indicate the type of supporting evidence: green (neighborhood), red (fusion), blue (co-occurrence), pink (experimentally determined), yellow-green (text mining), light blue (curated database), and black (coexpression), and purple (protein homology).

## 4 Discussion

Doxorubicin (DOX)-induced acute kidney injury (AKI) remains a significant limitation in cancer treatment, primarily due to its dose-dependent nephrotoxicity and delayed renal complications.^34^ These adverse effects are largely driven by oxidative stress, mitochondrial dysfunction, and activation of inflammatory pathways.^35,36^ Previous research has shown that nobiletin can reverse multidrug resistance in chemotherapy-resistant cancer cells by increasing intracellular DOX accumulation, suppressing the Nrf2 signaling pathway to reduce oxidative stress tolerance, and promoting p53-mediated apoptosis.^37,38^ Similarly, tangeretin has been reported to attenuate chemotherapy-induced oxidative stress and inflammation, leading to histological improvement in renal and other tissues, while enhancing antioxidant defenses.^39^ In this context, the current study aimed to investigate the potential of two polymethoxylated flavones–nobiletin and tangeretin–alongside the reference compound silymarin in alleviating DOX-induced nephrotoxicity.

One of the defining features of AKI is the accumulation of oxidative stress, which is characterized by enhanced lipid peroxidation, redox imbalance, and disruption of energy metabolism.^40^ Accordingly, DOX treatment in our study led to a marked increase in kidney weight and kidney-to-body weight ratio, along with a significant loss in overall body weight. These observations are consistent with earlier reports, which similarly linked these physiological alterations to oxidative damage and inflammatory responses.^29,30^ The significant decrease in serum MDA levels observed across all treatment groups indicates that each flavonoid effectively counteracted lipid peroxidation. Supporting this finding, Lahoti et al. (2012) reported increased serum urea and creatinine levels following DOX treatment, along with enhanced lipid peroxidation and reduced SOD activity in renal tissue.^35^

In response to oxidative injury, cells activate endogenous defense mechanisms, with NRF2 (nuclear factor erythroid 2-related factor 2) playing a central role in regulating antioxidant genes. Under basal conditions, NRF2 remains bound and suppressed by KEAP1 in the cytoplasm. ROS accumulation causes conformational changes in KEAP1, freeing NRF2 to translocate into the nucleus and induce the transcription of cytoprotective genes, such as *HO-1* expression.^41–43^ In the current study, DOX administration led to an expected elevation in *NRF2* and *HO-1* mRNA levels. While treatment groups exhibited a decrease in *NRF2* gene expression compared to the DOX group, these changes were not statistically significant, likely indicating a restored redox balance rather than an impaired response. Notably, HO-1 mRNA levels were partially suppressed in treatment groups; however, HO-1 protein expression remained elevated, likely due to its post-translational stability and extended half-life. This sustained expression suggests continued antioxidant protection at the protein level. Silymarin-treated rats exhibited persistently high HO-1 protein levels, consistent with its well-documented role in NRF2/HO-1 pathway activation. Nobiletin-treated rats demonstrated relatively lower HO-1 protein levels, though without statistical significance, possibly indicating restored redox homeostasis and reduced need for compensatory antioxidant response. Interestingly, *KEAP1* expression remained unaltered across all groups, suggesting potential KEAP1-independent regulation of NRF2, possibly driven by intense ROS accumulation induced by DOX. These observations align with the findings of Malik et al. (2015), who reported that nobiletin restored renal antioxidant enzyme levels and reduced oxidative stress in cisplatin-induced nephrotoxicity.^15^ Overall, the differential regulation of *NRF2* and *HO-1* by flavonoid treatments supports their capacity to modulate oxidative stress, either by sustaining antioxidant signaling (as observed with silymarin) or by promoting redox normalization (as observed with nobiletin).

In addition to oxidative damage, DOX-induced nephrotoxicity is closely associated with apoptotic cell death. Apoptosis, a programmed cell death pathway, is activated by DNA damage, oxidative insult, and disrupted signaling cascades. It involves both mitochondrial (intrinsic) and death receptor-mediated (extrinsic) pathways. In our study, DOX markedly upregulated several pro-apoptotic genes, including *BAX*, *CASP3*, *CASP8*, *CASP9*, *CYCS*, *PARP1*, and *TP53*, while downregulating the antiapoptotic gene *BCL2*, indicating concurrent activation of both apoptotic cascades. Caspase-3 protein levels were also significantly elevated, confirming the execution of apoptosis at the protein level. Among the treatment groups, nobiletin indicated the most pronounced suppression of *CASP3* expression, outperforming both tangeretin and silymarin. The statistically significant difference between nobiletin and tangeretin further supports nobiletin’s superior antiapoptotic efficacy. These findings are consistent with those of Lahoti et al. (2012), who reported a robust apoptotic response in DOX-treated kidneys, characterized by increased expression of *CASP3* and *BAX*, and reduced levels of *BCL-2* and *BCL-xL*. This molecular shift was accompanied by DNA fragmentation and severe histopathological damage, reinforcing the role of oxidative stress as a central trigger of both intrinsic and extrinsic apoptotic mechanisms in renal tissue.^35^ The apoptotic regulatory effect of nobiletin is further supported by the findings of Liu et al. (2018), who demonstrated that nobiletin increased *BAX* and *p53* expression, reduced *BCL-2* levels, and activated *CASP3* in MCF-7 cells, confirming its consistent role in modulating intrinsic apoptotic signaling.^38^

DOX also triggered a potent inflammatory response, as evidenced by increased expression of *TNF-*α, *IL-1*β, *IL-6*, *NF-*κ*B p65*, *AP-1*, and *p38 MAPK*, along with elevated TNF-α protein levels. Nobiletin and silymarin significantly suppressed *TNF-*α expression at both transcriptional and translational levels, whereas tangeretin’s effect appeared more selective, prominently reducing *CASP3* and *IL-6* while showing weaker suppression of *TNF-*α. All flavonoid-treated groups showed reduced expression of *NF-*κ*B* and *AP-1*, key transcription factors in cytokine regulation. Consistent with our findings, Lin et al. (2003) also demonstrated that nobiletin markedly inhibited LPS-induced *TNF-*α, *IL-1*α, *IL-1*β, and *IL-6* expression in macrophages, further supporting its anti-inflammatory action through interference with upstream signaling regulators like *NF-*κ*B* and *AP-1*. Furthermore, the decrease in *p38 MAPK* expression suggests interruption of upstream MAPK signaling, which may contribute to a broader inhibition of inflammatory pathways.^44^ Supporting this, Liu et al. (2018) reported that nobiletin enhanced p38 phosphorylation while suppressing NF-κB p65 nuclear translocation and NRF2 signaling in MCF-7 cells, further reinforcing its role in modulating key stress-responsive and inflammatory pathways.^38^ In a TNBS-induced colitis mouse model, oral tangeretin treatment led to a noticeable decrease in *TNF-*α expression and a clear attenuation of NF-κB and MAPK pathway activation within the inflamed colon tissue. These molecular changes were accompanied by marked improvements in tissue morphology, pointing to tangeretin’s capacity to mitigate inflammatory damage *in vivo*. Taken together with the present findings, this supports the view that tangeretin can effectively interfere with TNF-α-driven inflammatory signaling and suppress NF-κB/MAPK activity under pathological conditions.^23^ Similar regulatory effects of tangeretin were also observed in a cisplatin-induced liver injury model, where it reduced TNF-α and phospho-p38 levels, lowered MDA and *NRF2* expression, and restored antioxidant defenses such as GSH and BCL-2, highlighting its multifaceted protective role against oxidative and inflammatory damage.^45^ A comparable inhibitory effect of nobiletin was also demonstrated in a diabetic nephrotoxicity model, where it suppressed *NF-*κ*B p65* and *BAX* expression while enhancing *BCL-2* levels, further substantiating its dual anti-inflammatory and antiapoptotic role in renal tissue.^46^

In the present study, in addition to NF-κB and p38 MAPK modulation, other transcription factors such as *ERK1/2 (MAPK3)* were also affected. DOX significantly induced *ERK1/2* expression, which was effectively reduced by silymarin, while nobiletin and tangeretin showed nonsignificant downward trends. Considering ERK’s involvement in cell proliferation, apoptosis, and inflammation, its suppression suggests that silymarin may exert broader regulatory effects on cellular stress responses.^47^ Particularly notable was the more than 50-fold reduction in *IL-1*β expression, reflecting a highly potent anti-inflammatory response across all treatments, which may help prevent the transition from acute to chronic inflammation. These transcriptional modulations are consistent with previous evidence supporting the multitargeted anti-inflammatory actions of polymethoxylated flavones.

Our histological findings in the DOX group confirmed severe renal injury, characterized by tubular necrosis and interstitial inflammation, consistent with the upregulation of apoptotic and inflammatory genes. Notably, treatment groups–particularly those receiving nobiletin–exhibited substantially improved renal morphology, supported by lower damage scores and reduced *CASP3*, *PARP1*, and *TNF-*α expression. Furthermore, the treatments significantly limited fibrotic progression, indicating a robust tissue-protective effect.^47,48^

Molecular docking analyses provided further insights into compound–target interactions. Silymarin showed the most favorable binding affinities (more negative binding energies) across all selected targets, including CASP3, TNF-α, HO-1, NF-κB, and IL-6. However, *in vivo* results–encompassing gene and protein expression data, as well as histological outcomes–consistently favored nobiletin. This suggests that while silymarin indicates strong *in silico* affinity, its limited absorption and high polarity may reduce bioavailability and systemic effectiveness. Pharmacokinetic simulations further supported this interpretation: nobiletin and tangeretin were predicted to have high gastrointestinal absorption and favorable permeability parameters (iLOGP > 3.7; TPSA < 90 Å^2^), whereas silymarin exhibited poor absorption (TPSA = 155.14 Å^2^) and fell outside BOILED-EGG’s predicted absorption zones. In terms of CYP inhibition, nobiletin and tangeretin were identified as dual inhibitors of CYP2C9 and CYP3A4, while silymarin only inhibited CYP3A4. Although this raises the possibility of drug–drug interactions, it may also enhance the metabolic stability of nobiletin in therapeutic settings. These pharmacokinetic distinctions explain the disparity between molecular docking predictions and *in vivo* efficacy. Nobiletin, along with the other compounds, demonstrated favorable bioavailability, modulation of gene and protein expression, and protective effects in histological analyses.

To better understand the interplay among targeted genes, a protein–protein interaction (PPI) network was constructed. The resulting network, comprising 14 nodes and 87 edges, supported a highly significant degree of connectivity (*p* < 1.0e–16). Central hub proteins included CASP3, BAX, MAPK3 (representing ERK1/2 and p38 MAPK), LOC103694380 (membrane-bound TNF-α form), IL6, and TP53, which are key regulators of apoptosis, oxidative defense, and inflammation. The network underscores the interconnected nature of these molecular pathways and supports the multitargeted actions of the flavonoids studied. When integrated with molecular data, protein expression profiles, docking results, and histopathological findings, the PPI network further affirms the therapeutic potential of nobiletin, tangeretin, and silymarin in mitigating DOX-induced renal injury. In addition, existing literature reports support the notion that both nobiletin and tangeretin can form stable complexes with their respective protein targets under physiological conditions, as evidenced by molecular dynamics (MD) simulations. Nobiletin has been shown to bind to the p53 protein spontaneously, maintaining conformational stability over a 50 ns simulation with low RMSD fluctuations and favorable binding free energy. Similarly, tangeretin exhibited RMSD and RMSF profiles comparable to diazepam when bound to the GABAA receptor α3 subunit during a 50 ns MD simulation. Furthermore, both flavonoids preserved structural integrity and binding stability with the β-lactoglobulin transport protein in 10 ns MD simulations. Collectively, these findings provide strong external validation for the physiological relevance of our docking results.^37,39,49^

Finally, despite the comprehensive nature of this study, certain limitations remain. *In silico* pharmacokinetic predictions should be validated through experimental absorption and distribution studies. Moreover, additional analyses–such as intracellular ROS quantification, caspase activity assays, and mitochondrial membrane potential assessments–would further strengthen the mechanistic insights. In addition, molecular dynamics (MD) simulations were not performed in the present study. While literature-based MD data provide supportive evidence for the stability of nobiletin and tangeretin interactions with relevant protein targets, future in-house MD simulations will be essential to confirm binding stability under physiological conditions and to complement our docking analyses. Taken together, these findings suggest that nobiletin demonstrated a trend toward a broader and more consistent protective profile than the other flavonoids tested, while all three compounds showed notable nephroprotective potential. Its favorable pharmacokinetics may help bridge the gap between molecular affinity and *in vivo* efficacy, supporting its consideration for further preclinical development.

## 5 Conclusion

This study indicates that nobiletin, tangeretin, and silymarin exhibit significant nephroprotective effects against DOX-induced renal injury by modulating oxidative stress, apoptosis, and inflammation. While all compounds provided comparable biochemical and histological improvements, nobiletin exhibited a consistent multilevel protective profile, with notable modulation of key apoptotic and inflammatory markers compared to tangeretin. Although silymarin showed superior docking affinity, its limited bioavailability reduced its *in vivo* potential. These findings position nobiletin as a strong candidate for further development as a nephroprotective agent, necessitating further development via advanced formulations and long-term therapeutic evaluations.

However, this study has certain limitations. First, the relatively small sample size, while sufficient to reveal significant molecular and histological changes, may restrict the broader applicability of the results. Second, only male rats were used, consistent with standard practice in DOX-induced AKI models to minimize hormonal variability; nevertheless, potential sex-specific responses cannot be excluded. Lastly, although this study conducted detailed molecular analyses, it did not include broader functional assays such as enzymatic activity measurements, oxidative stress biomarkers, or renal function parameters. Incorporating such functional evaluations in future studies would enhance the translational value of the findings. Finally, molecular dynamics simulations were not performed; although external literature reports provide supportive MD evidence for the stability of nobiletin and tangeretin complexes, future in-house MD analyses will be valuable to validate the docking predictions and strengthen mechanistic insights.

## Supplementary Material



## Notes

Docking-based interaction profiles of nobiletin, tangeretin, and silymarin against apoptosis, inflammation, and oxidative stress targets (Table S1); docking results were obtained using CB-Dock2 and evaluated in terms of predicted binding affinity and contact residues; color coding in the docking images: hydrogen bonds (blue lines), hydrophobic interactions (green lines), electrostatic or polar interactions (red lines); residue lists provided correspond to those within the predicted binding pockets; full interaction maps and PDB identifiers alongside the images (PDF)
