# Supplementary material for: Nobiletin Ameliorates Doxorubicin-Induced Nephrotoxicity by Targeting Oxidative Stress, Inflammatory, and Apoptotic Pathways: Combined In Vivo and In Silico Insights
Source: ACS Omega. 2026 Jan 23;11(5):7165–77. doi: 10.1021/acsomega.5c07049 (PMC12902976; doi:10.1021/acsomega.5c07049)
Supplement: Supplementary file 1 [file ao5c07049_si_001.pdf]

Supporting information

Manuscript ID: ao-2025-070498.R2

Nobiletin Ameliorates Doxorubicin-Induced Nephrotoxicity by Targeting Oxidative Stress, Inflammatory, and Apoptotic Pathways: Combined *In Vivo* and *In Silico* Insights

Sümeyra Çetinkaya[1], İpek Süntar[2,*], Mürşide Ayşe Demirel[3], İlknur Çınar Ayan[4,*], Özen Akarca Dizakar[5]

[1]*Biotechnology Research Center, Field Crops Central Research Institute, Ankara 06170, Türkiye*
[2]*Department of Pharmacognosy, Faculty of Pharmacy, Gazi University, Ankara 06630, Türkiye*
[3]*Department of Basic Pharmaceutical Sciences, Faculty of Pharmacy, Gazi University, Ankara 06630, Türkiye*
[4]*Department of Medical Biology, Faculty of Medicine, Necmettin Erbakan University, Konya 42090, Türkiye*
[5]*Department of Histology and Embryology, Faculty of Medicine, Bakırçay University, İzmir 35665, Türkiye*

Table S1. Docking-based interaction profiles of nobiletin, tangeretin and silymarin against apoptosis, inflammation and oxidative stress targets. Docking results were obtained using CB-Dock2 and evaluated in terms of predicted binding affinity and contact residues. Color coding in the docking images: hydrogen bonds (blue lines), hydrophobic interactions (green lines), electrostatic or polar interactions (red lines). The residue lists provided correspond to those within the predicted binding pockets. Full interaction maps and PDB identifiers are included alongside the images.

Targets with PDB identifier	Contact Residues (Nobiletin versus targets)	
BAX (6EB6)	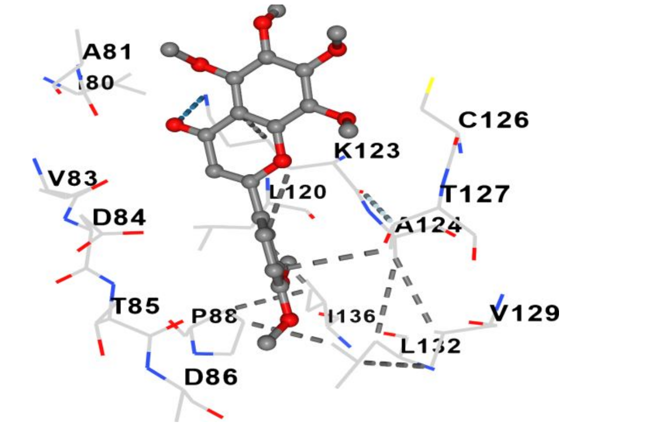	**Chain A**: ILE80 ALA81 ALA82 VAL83 ASP84 THR85 ASP86 SER87 PRO88 GLU90 ARG94 LEU120 LYS123 ALA124 CYS126 THR127 VAL129 LEU132 ILE136 TRP188 LYS189 LYS190 MET191
BCL-2 (1G5M)	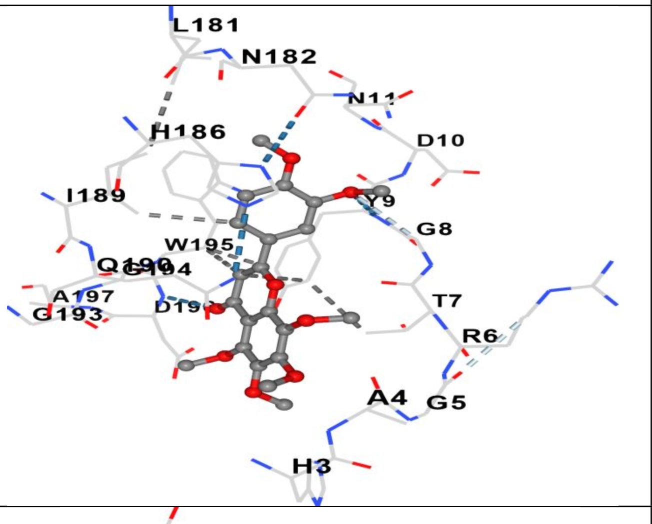	**Chain A**: HIS3 ALA4 GLY5 ARG6 THR7 GLY8 TYR9 ASP10 ASN11 LEU181 ASN182 HIS186 ILE189 GLN190 GLY193 GLY194 TRP195 ASP196
CASP3 (6CKZ)	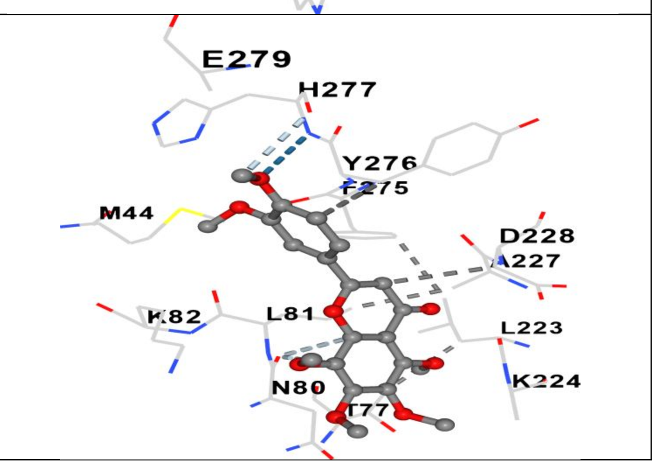	**Chain A**: MET39 ASP40 TYR41 PRO42 GLU43 MET44 ARG75 THR77 ARG79 ASN80 LEU81 LYS82 TYR83 GLU84 VAL85 ASN87<br>**Chain B**: LEU223 LYS224 ALA227 ASP228 PHE275 TYR276 HIS277 GLU279

<table>
<tr><td>PARP-1 (4DQY)</td><td>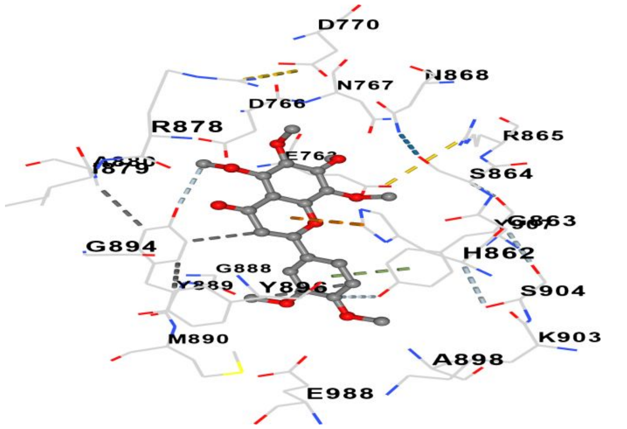</td><td>Chain C: LYS703 TYR710 GLN759 GLU763 ASP766 ASN767 LEU769 ASP770 HIS862 GLY863 SER864 ARG865 ASN868 ILE872 LEU877 ARG878 ILE879 ALA880 PRO881 THR887 GLY888 TYR889 MET890 GLY894 ILE895 TYR896 PHE897 ALA898 LYS903 SER904 TYR907 HIS909 GLU988</td></tr>
<tr><td>p53 (1TUP)</td><td>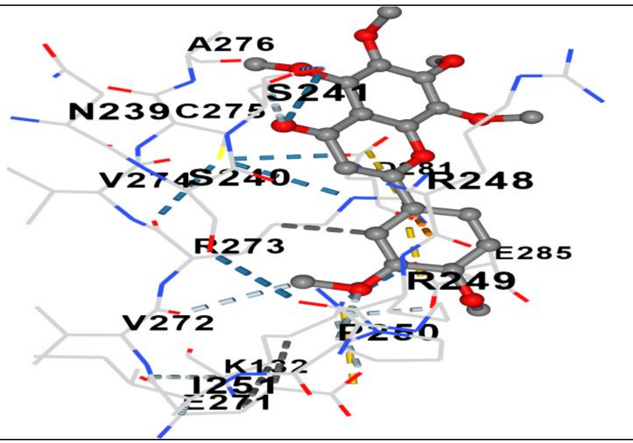</td><td>Chain C: LYS120 LYS132 ASN239 SER240 SER241 ARG248 ARG249 PRO250 ILE251 GLU271 VAL272 ARG273 VAL274 CYS275 ALA276 ARG280 ASP281 GLU285</td></tr>
<tr><td>CYCS (3ZCF)</td><td>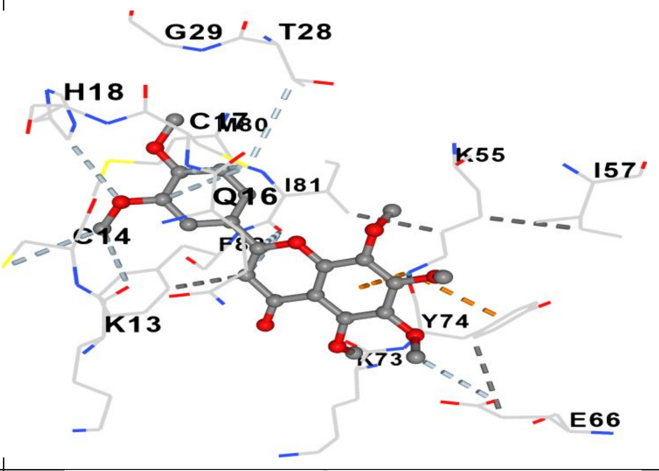</td><td>Chain B: MET12 LYS13 CYS14 GLN16 CYS17 HIS18 THR28 GLY29 PRO30 TYR46 TYR48 THR49 ASN52 TYR67 THR78 LYS79 MET80 ILE81 PHE82 VAL83 GLY84
Chain C: ASN54 LYS55 GLY56 ILE57 THR63 GLU66 LYS73 TYR74 ILE75 PRO76</td></tr>
<tr><td>NRF2 (5WFV)</td><td>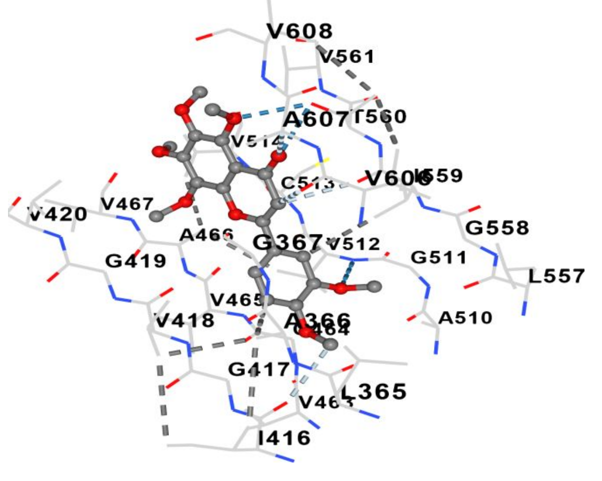</td><td>Chain A: GLY364 LEU365 ALA366 GLY367 CYS368 VAL369 ARG415 ILE416 GLY417 VAL418 GLY419 VAL420 GLY462 VAL463 GLY464 VAL465 ALA466 VAL467 GLY509 ALA510 GLY511 VAL512 CYS513 VAL514 ALA556 LEU557 GLY558 ILE559 THR560 VAL561 GLY603 VAL604 GLY605 VAL606 ALA607 VAL608</td></tr>
</table>

KEAP1 (8X34)	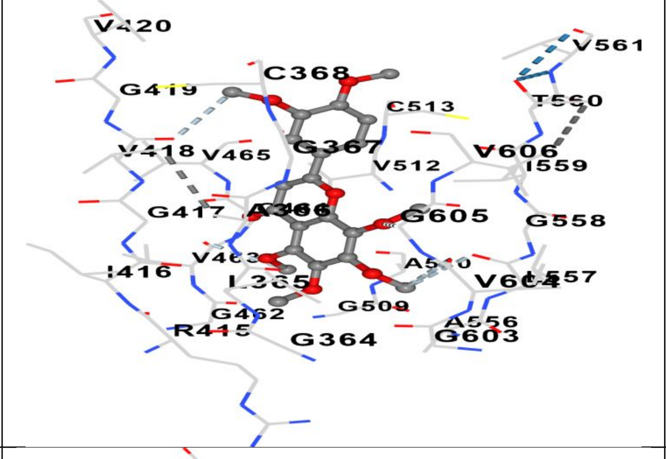	**Chain B**: TYR334 SER363 GLY364 LEU365 ALA366 GLY367 CYS368 ASN382 ASN414 ARG415 ILE416 GLY417 VAL418 GLY419 VAL420 GLY462 VAL463 GLY464 VAL465 ALA466 VAL467 GLY509 ALA510 GLY511 VAL512 CYS513 VAL514 SER555 ALA556 LEU557 GLY558 ILE559 THR560 VAL561 TYR572 PHE577 SER602 GLY603 VAL604 GLY605 VAL606 ALA607 VAL608ASP385 ASN387 THR388 ASP389 SER390 SER391 ALA392 PRO412
HO-1 (1N3U))	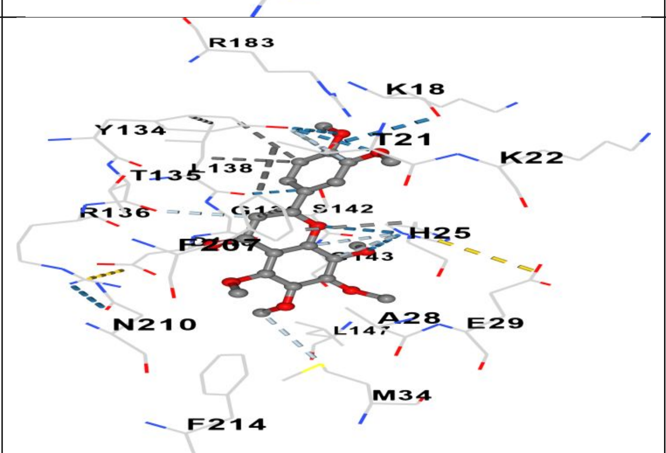	**Chain B**: LYS18 THR21 LYS22 HIS25 ALA28 GLU29 MET34 GLN38 VAL50 LEU54 TYR134 THR135 ARG136 LEU138 GLY139 ASP140 SER142 GLY143 VAL146 LEU147 ILE150 LYS179 ARG183 PHE207 ASN210 PHE214
TNF-α (1TNF)	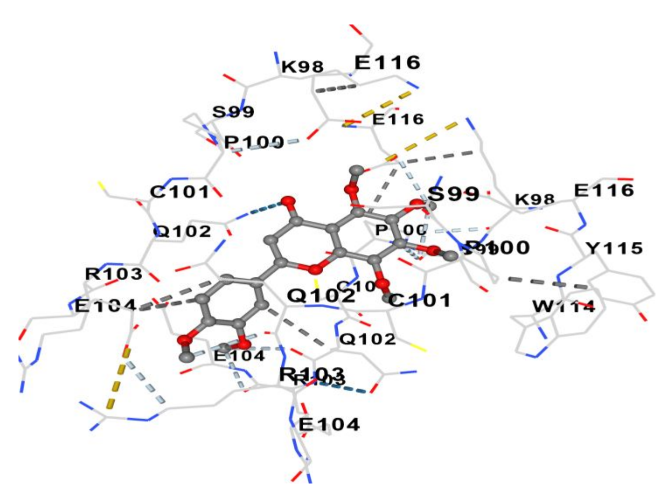	**Chain A**: SER99 PRO100 CYS101 GLN102 ARG103 GLU104 THR105 TRP114 TYR115 GLU116 **Chain B**: LYS98 SER99 PRO100 CYS101 GLN102 ARG103 GLU104 THR105 GLU116 **Chain C**: CYS69 LYS98 SER99 PRO100 CYS101 GLN102 ARG103 GLU104 TYR115 GLU116
IL-1β (1I1B)	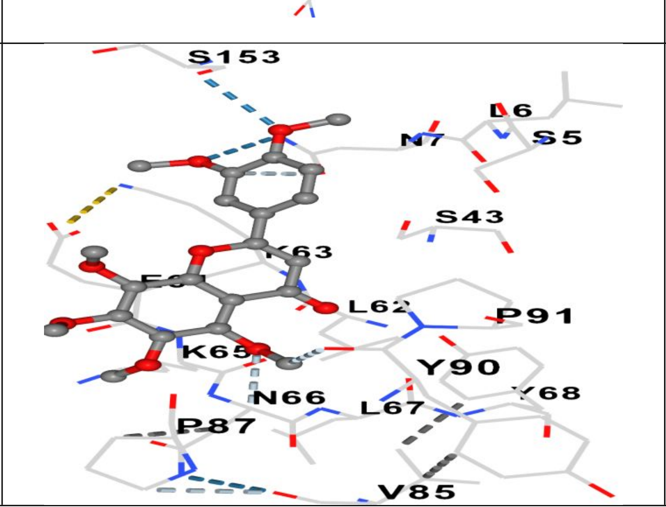	**Chain A**: VAL3 ARG4 SER5 LEU6 ASN7 SER43 SER45 GLY61 LEU62 LYS63 GLU64 LYS65 ASN66 LEU67 TYR68 VAL85 ASP86 PRO87 LYS88 TYR90 PRO91 SER152 SER153

IL-6 (1ALU)	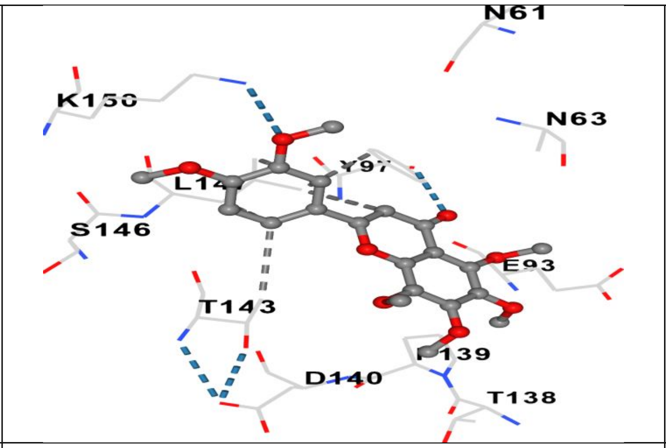	**Chain A**: ASN61 ASN63 LEU64 PRO65 THR89 LEU92 GLU93 GLU95 VAL96 TYR97 GLU99 GLN116 LYS120 ILE123 GLN127 ILE136 THR137 THR138 PRO139 ASP140 PRO141 THR142 THR143 ASN144 ALA145 SER146 LEU147 LEU148 LYS150
NF-κβ p65 (4G3D)	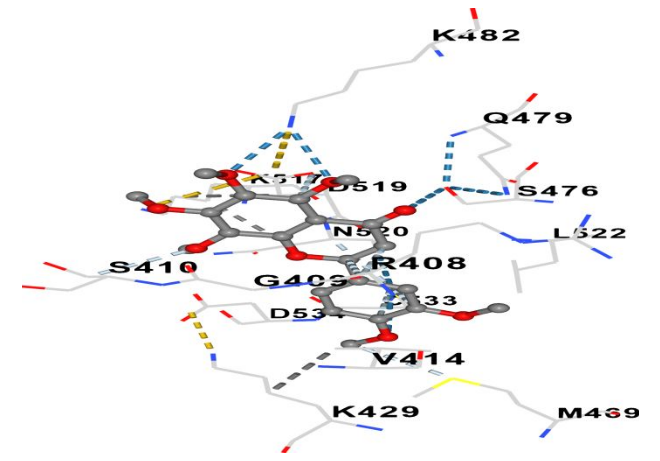	**Chain E**: HIS402 GLY407 ARG408 GLY409 SER410 GLU413 VAL414 HIS415 ARG416 ALA427 LYS429 VAL453 MET469 GLU470 LEU471 LEU472 GLU473 GLY474 GLY475 SER476 GLN479 LYS482 LYS517 ASP519 ASN520 LEU522 CYS533 ASP534
p38 MAPK (2Y8O)	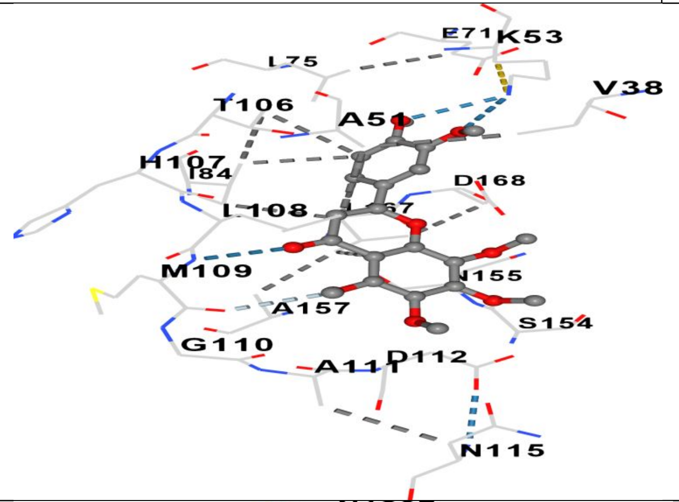	**Chain A**: VAL30 GLY31 SER32 TYR35 VAL38 ALA51 LYS53 ARG67 GLU71 LEU75 ILE84 LEU86 LEU104 VAL105 THR106 HIS107 LEU108 MET109 GLY110 ALA111 ASP112 ASN115 ASP150 LYS152 SER154 ASN155 LEU156 ALA157 LEU167 ASP168 PHE169 GLY170 LEU171 ALA172
CASP9 (5JUY)	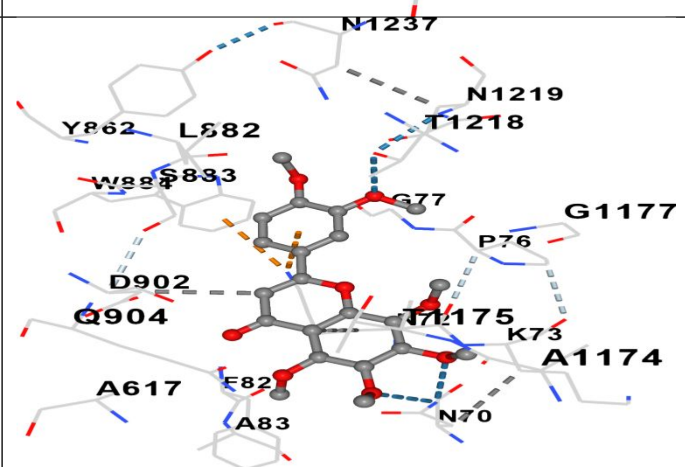	**Chain D**: THR615 ASP616 ALA617 ALA635 TYR862 LEU882 SER883 TRP884 ASP902 GLN904 GLN924 LEU1089 SER1090 ARG1131 ASP1147 ASN1148 ALA1173 ALA1174 THR1175 GLY1177 GLY1178 TRP1179 VAL1180 THR1181 ASP1182 THR1218 ASN1219 LYS1221 LYS1222 ASN1237<br>**Chain K**: ASP50 LYS53 ASN54 ASN70 LYS72 LYS73 PRO76 GLY77 THR78 ILE81 PHE82 ALA83

CASP8 (6PX9)	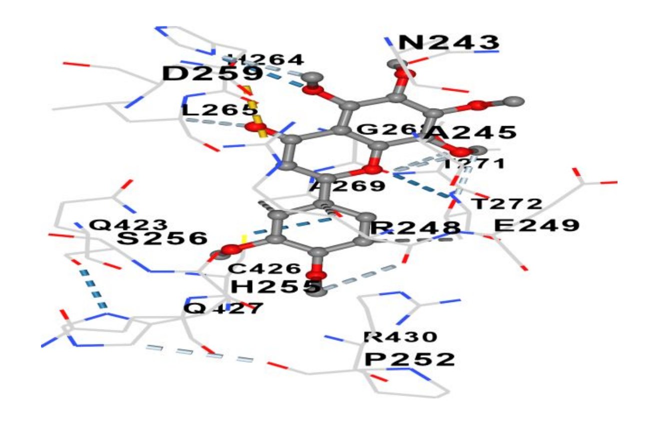	**Chain B**: ASN243 ALA245 ARG248 GLU249 VAL251 PRO252 LYS253 LEU254 HIS255 SER256 ILE257 ARG258 ASP259 ASN261 HIS317 GLY318 ASP319 TYR324 CYS360 GLN361 **Chain D**: HIS264 LEU265 ASP266 GLY268 ALA269 THR271 THR272 THR273 TRP420 GLN423 SER424 CYS426 GLN427 ARG430
AP-1 (4HMY)	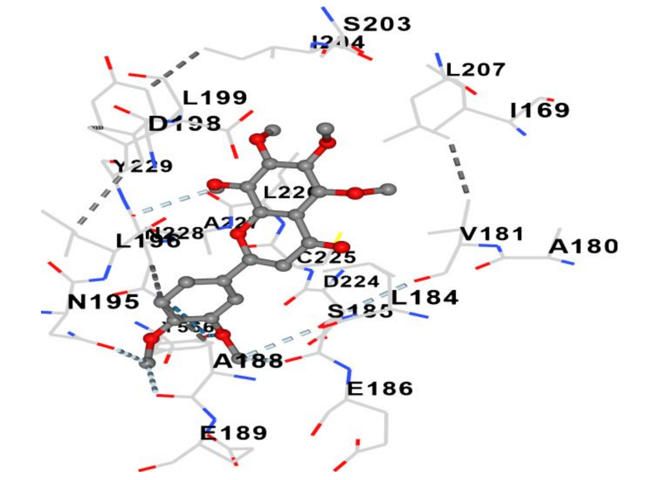	**Chain B**: ILE169 ALA180 VAL181 LEU184 SER185 GLU186 ALA188 GLU189 HIS191 ASN195 LEU196 ASP198 LEU199 SER203 ILE204 LEU207 PHE221 ASP224 CYS225 LEU226 ALA227 ASN228 TYR229 TYR566 THR569 **Chain M**: SER2 ASN74 ALA75 CYS76 TYR122
**Targets**	**Contact Residues (Tangeretin versus targets)**	
BAX (6EB6)	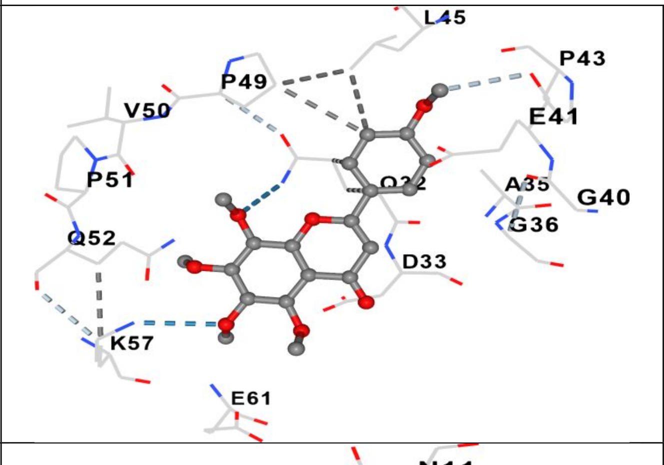	**Chain A**: GLY29 GLN32 ASP33 ALA35 GLY36 ARG37 GLY40 GLU41 PRO43 LEU45 PRO49 VAL50 PRO51 GLN52 LYS57 SER60 GLU61 LYS64
BCL-2 (1G5M)	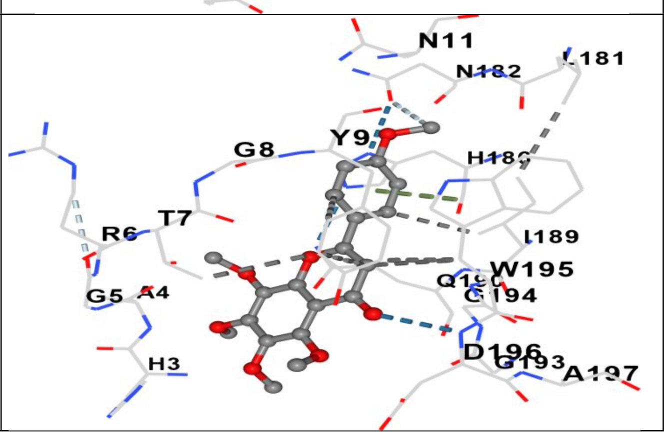	**Chain A**: HIS3 ALA4 GLY5 ARG6 THR7 GLY8 TYR9 ASP10 ASN11 LEU181 ASN182 HIS186 ILE189 GLN190 GLY193 GLY194 TRP195 ASP196 ALA197

CASP3 (6CKZ)	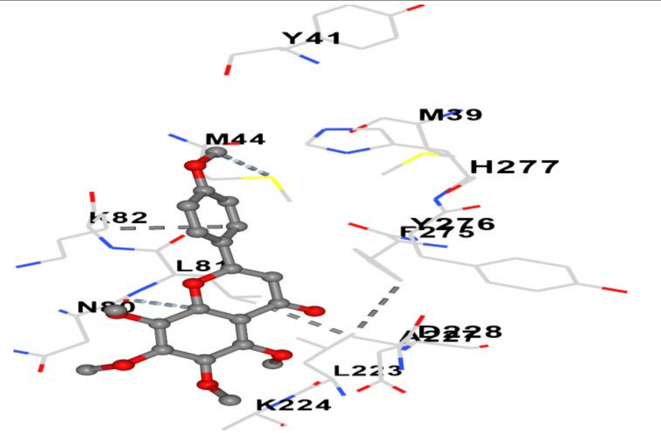	**Chain A**: MET39 ASP40 TYR41 PRO42 GLU43 MET44 ARG75 ARG79 ASN80 LEU81 LYS82 TYR83 GLU84 VAL85 **Chain B**: LEU223 LYS224 ALA227 ASP228 PHE275 TYR276 HIS277 GLU279
PARP-1 (4DQY)	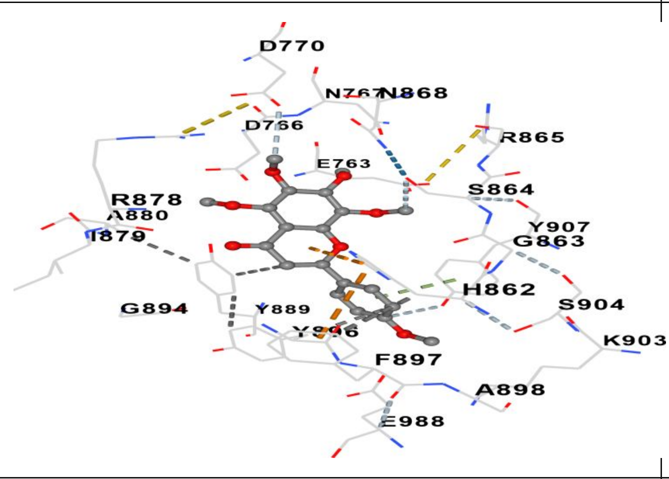	**Chain C**: LYS703 TYR710 GLN759 ALA762 GLU763 ASP766 ASN767 LEU769 ASP770 VAL773 HIS862 GLY863 SER864 ARG865 ASN868 ILE872 GLN875 GLY876 LEU877 ARG878 ILE879 ALA880 PRO881 THR887 GLY888 TYR889 GLY894 ILE895 TYR896 PHE897 ALA898 LYS903 SER904 TYR907 HIS909 GLU988
p53 (1TUP)	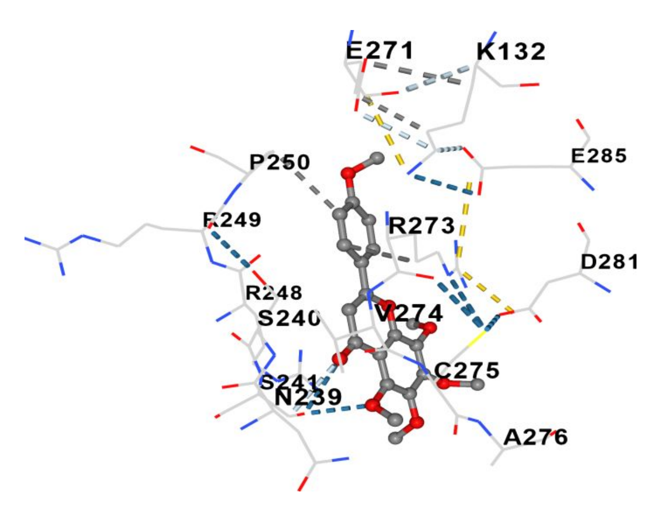	**Chain C**: LEU130 LYS132 LYS164 GLN165 ASN239 SER240 SER241 ARG248 ARG249 PRO250 GLU271 VAL272 ARG273 VAL274 CYS275 ALA276 ASP281 GLU285 ASN288 LEU289
CYCS (3ZCF)	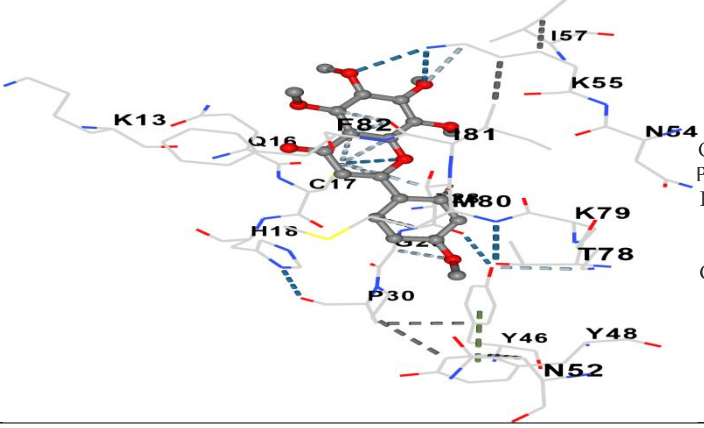	**Chain B**: MET12 LYS13 CYS14 GLN16 CYS17 HIS18 THR28 GLY29 PRO30 TYR46 TYR48 ASN52 THR78 LYS79 MET80 ILE81 PHE82 VAL83 GLY84 **Chain C**: LYS39 ASN54 LYS55 GLY56 ILE57 THR63 GLU66 LYS73 TYR74

NRF2 (5WFV)	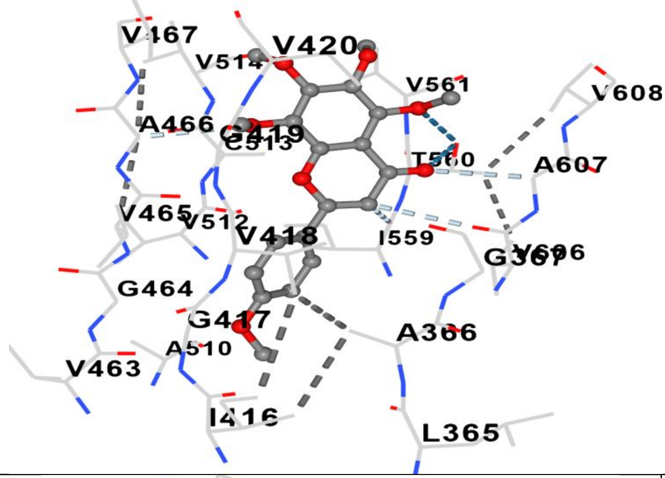	**Chain A**: GLY364 LEU365 ALA366 GLY367 CYS368 VAL369 ARG415 ILE416 GLY417 VAL418 GLY419 VAL420 GLY462 VAL463 GLY464 VAL465 ALA466 VAL467 GLY509 ALA510 VAL512 CYS513 VAL514 ALA556 LEU557 GLY558 ILE559 THR560 VAL561 GLY603 VAL604 GLY605 VAL606 ALA607 VAL608
KEAP1 (8X34)	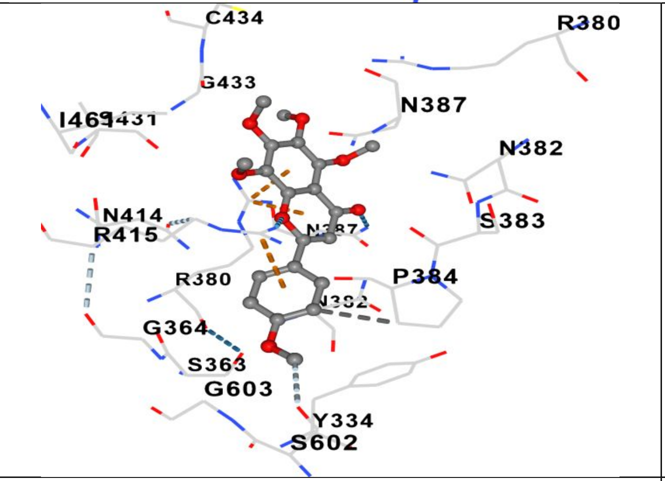	**Chain A**: TYR334 SER363 GLY364 ARG380 ASN382 SER383 PRO384 GLY386 ASN387 ASN414 ARG415 SER431 HIS432 GLY433 CYS434 ILE435 HIS436 ILE461 SER602 GLY603 **Chain B**: TYR334 ARG336 SER363 GLY364 ARG380 ASN382 SER383 PRO384 ASN387 ASN414 ARG415 GLY433 TYR525 GLN530 SER555 ALA556 TYR572 PHE577 SER602 GLY603
HO-1 (1N3U))	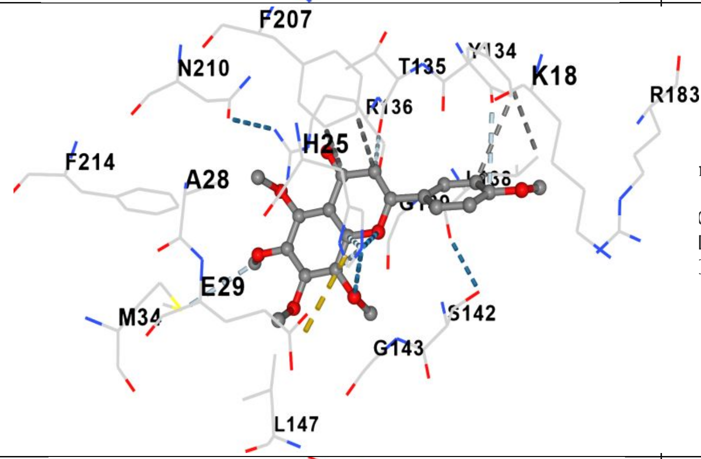	[illegible]ain A: SER14 LYS18 THR21 HIS25 ALA28 GLU29 MET34 ARG35 GLN38 TYR134 THR135 ARG136 LEU138 GLY139 ASP140 SER142 GLY143 LEU147 LYS179 ARG183 PHE207 ASN210 PHE214
TNF-α (1TNF)	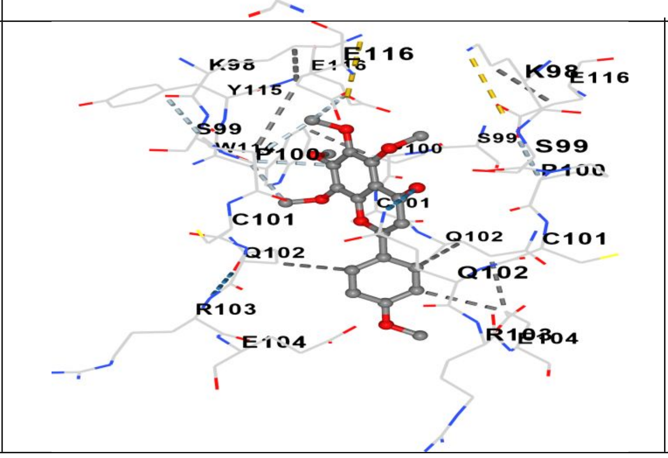	**Chain A**: LYS98 SER99 PRO100 CYS101 GLN102 ARG103 TRP114 TYR115 GLU116 **Chain B**: LYS98 SER99 PRO100 CYS101 GLN102 ARG103 GLU104 GLU116 **Chain C**: LYS98 SER99 PRO100 CYS101 GLN102 ARG103 GLU104 GLU116

IL-1β (1I1B)	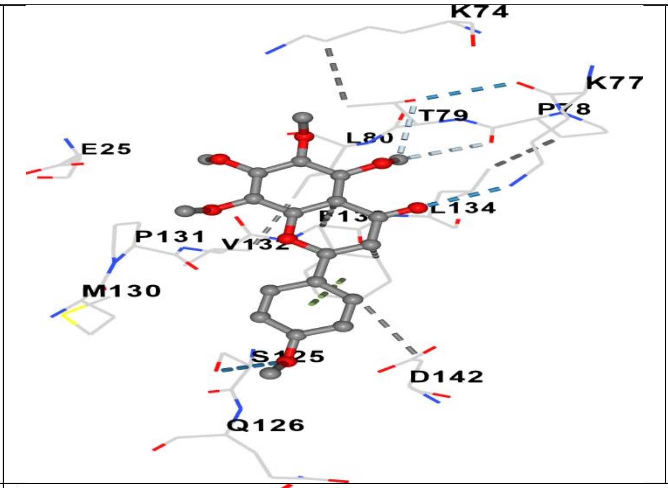	**Chain A**: PRO23 TYR24 GLU25 LYS74 ASP75 LYS77 PRO78 THR79 LEU80 GLN81 LEU82 GLU83 SER84 SER125 GLN126 MET130 PRO131 VAL132 PHE133 LEU134 GLY135 ASP142
IL-6 (1ALU)	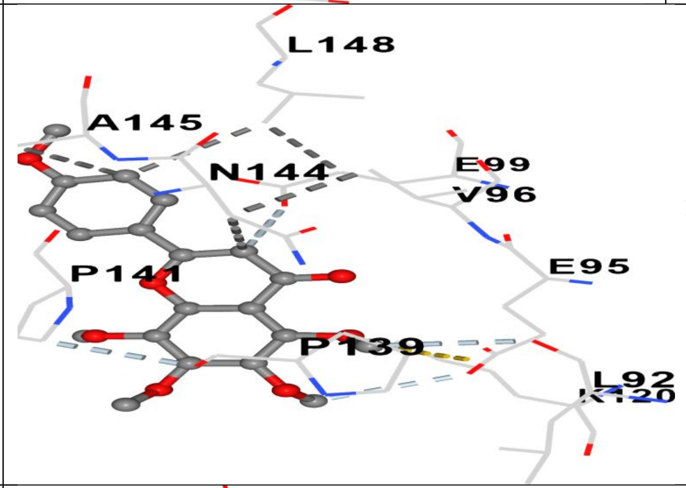	**Chain A**: ASN61 LEU62 ASN63 LEU64 PRO65 THR89 LEU92 GLU93 GLU95 VAL96 TYR97 GLU99 LYS120 THR137 THR138 PRO139 ASP140 PRO141 THR143 ASN144 ALA145 LEU147 LEU148 LYS150
NF-κβ p65 (4G3D)	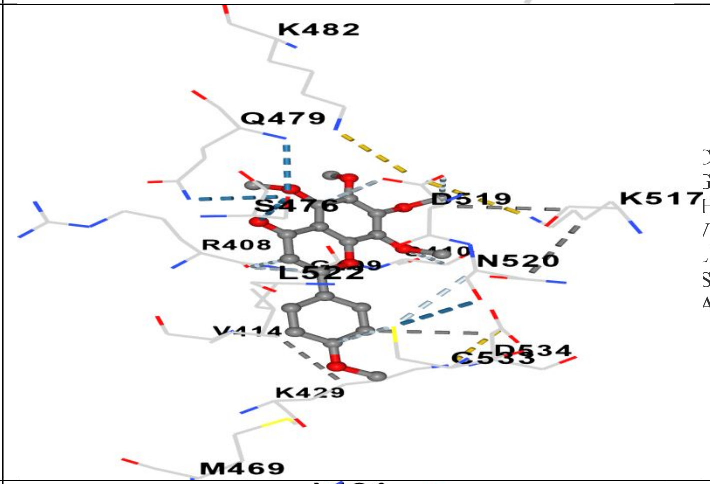	**hain E**: HIS402 GLY407 ARG408 LY409 SER410 GLU413 VAL414 HIS415 ARG416 ALA427 LYS429 AL453 MET469 GLU470 LEU471 EU472 GLU473 GLY474 GLY475 SER476 GLN479 LYS482 LYS517 ASP519 ASN520 LEU522 CYS533 ASP534
p38 MAPK (2Y8O)	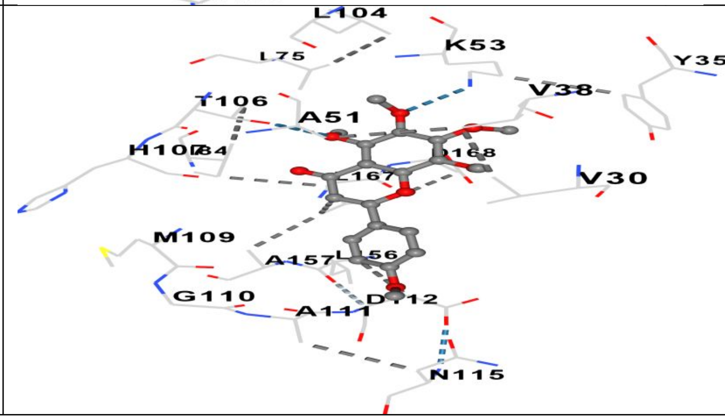	**hain A**: VAL30 GLY31 SER32 35 VAL38 ALA51 LYS53 ARG67 71 LEU74 LEU75 ILE84 LEU104 R106 HIS107 LEU108 MET109 Y110 ALA111 ASP112 ASN115 S152 SER154 ASN155 LEU156 A157 LEU167 ASP168 PHE169 GLY170 LEU171 ALA172

CASP9 (5JUY)	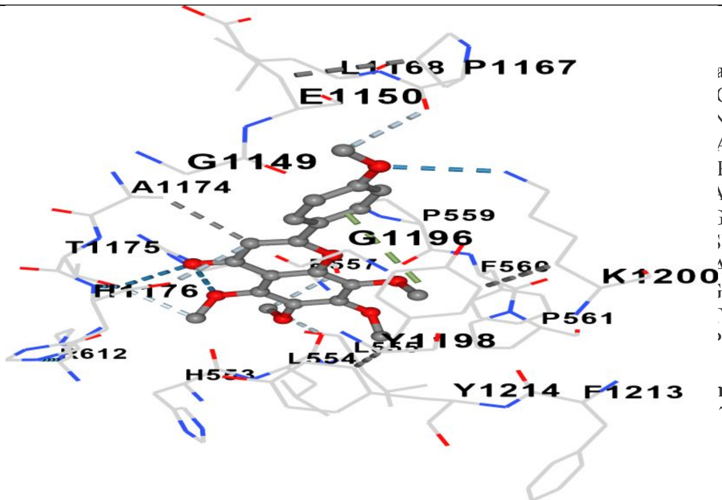	**ain F**: HIS553 LEU554 LEU555 G557 PRO559 PHE560 PRO561 N565 ARG612 ASP616 ALA617 A635 TYR862 LEU882 SER883 P884 ASP902 ASP903 GLN904 ASP1147 ASN1148 GLY1149 GLU1150 PRO1167 LEU1168 ER1169 GLY1172 ALA1173 ALA1174 THR1175 HIS1176 GLY1177 GLY1178 GLY1196 TYR1198 LYS1200 GLN1211 PHE1213 TYR1214 THR1218 ASN1219 ASN1237 **ain M**: ASN70 LYS72 LYS73 76 GLY77 ILE81 PHE82 ALA83 LYS86
CASP8 (6PX9)	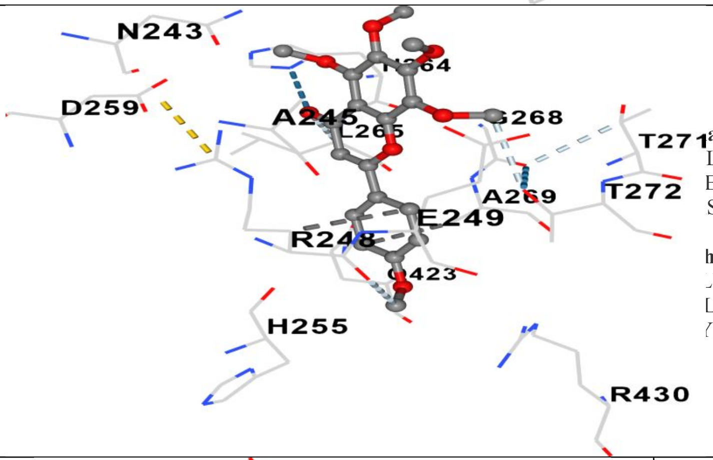	**ain B**: ASN243 ALA245 ARG248 LU249 LYS253 LEU254 HIS255 ER256 ILE257 ARG258 ASP259 SN261 HIS317 ASP319 TYR324 CYS360 GLN361 **hain D**: HIS264 LEU265 ASP266 Y268 ALA269 THR271 THR272 LU275 TRP420 GLN423 SER424 YS426 GLN427 ARG430 GLU449
AP-1 (4HMY)	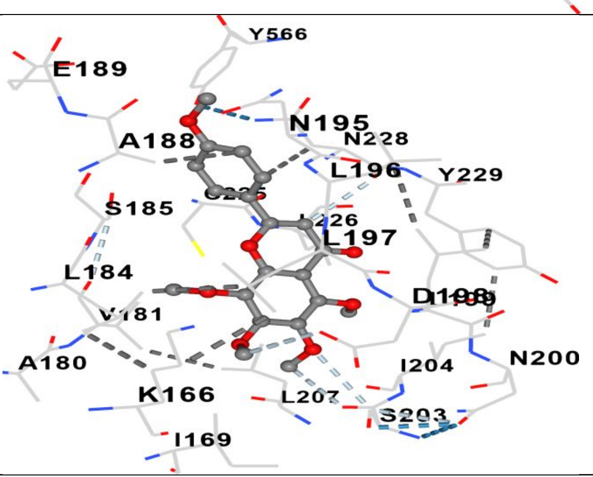	**Chain B**: LYS166 ILE169 ALA180 VAL181 LEU184 SER185 ALA188 GLU189 ASN195 LEU196 LEU197 ASP198 LEU199 ASN200 SER203 ILE204 LEU207 LEU208 CYS225 LEU226 ALA227 ASN228 TYR229 PRO231 GLU236 ILE240 PHE266 TYR566
**Targets**	**Contact Residues (Silymarin versus targets)**	
BAX (6EB6)	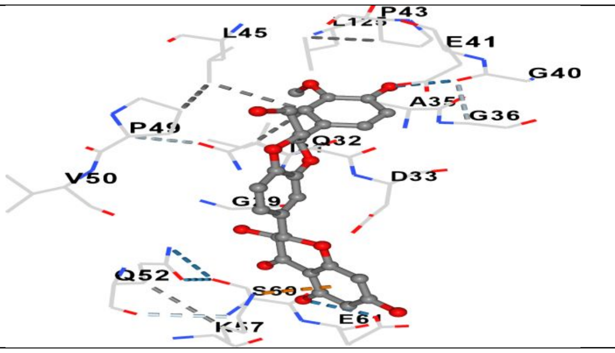	**Chain A**: LEU25 GLN28 GLY29 PHE30 ILE31 GLN32 ASP33 ARG34 ALA35 GLY36 ARG37 GLY40 GLU41 ALA42 PRO43 GLU44 LEU45 ASP48 PRO49 VAL50 PRO51 GLN52 ASP53 THR56 LYS57 LYS58 SER60 GLU61 LYS64 ASP68 LEU125

BCL-2 (1G5M)	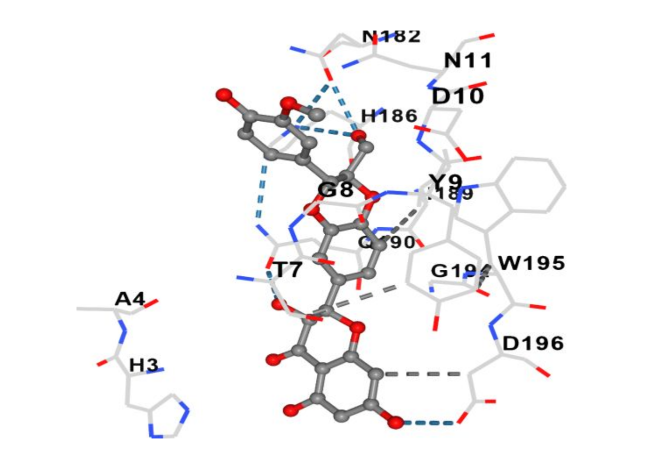	**Chain A**: HIS3 ALA4 GLY5 ARG6 THR7 GLY8 TYR9 ASP10 ASN11 ILE14 LEU181 ASN182 HIS186 ILE189 GLN190 ASP191 ASN192 GLY193 GLY194 TRP195 ASP196 ALA197
CASP3 (6CKZ)	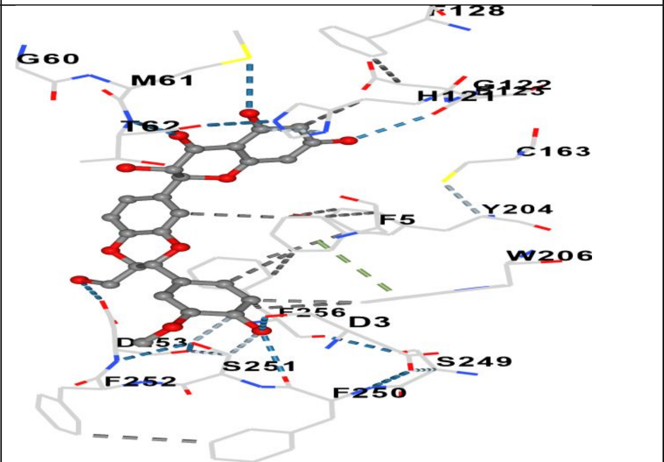	**Chain A**: THR59 GLY60 MET61 THR62 SER63 ARG64 SER65 HIS121 GLY122 GLU123 GLU124 PHE128 CYS163 ARG164 GLY165 THR166 **Chain B**: TYR204 TRP206 ARG207 SER209 SER249 PHE250 SER251 PHE252 ASP253 PHE256 **Chain C**: ASP3 PHE5
PARP-1 (4DQY)	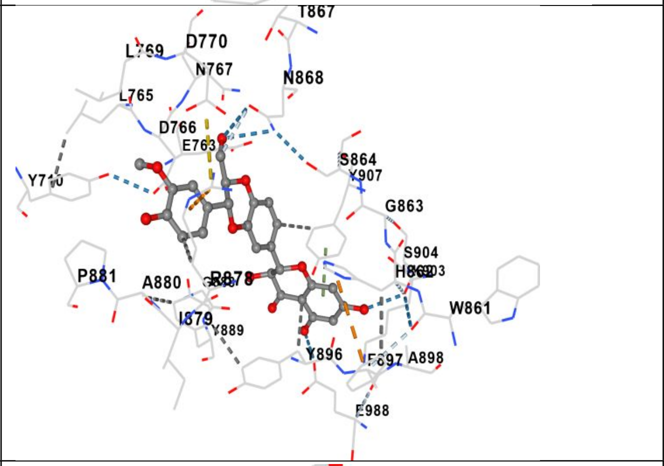	**Chain C**: TYR689 LYS703 ILE706 GLN707 TYR710 GLN759 ALA760 GLU763 LEU765 ASP766 ASN767 LEU769 ASP770 VAL773 TRP861 HIS862 GLY863 SER864 ARG865 THR867 ASN868 ILE872 GLN875 LEU877 ARG878 ILE879 ALA880 PRO881 PRO882 GLU883 THR887 GLY888 TYR889 GLY894 ILE895 TYR896 PHE897 ALA898 LYS903 SER904 ASN906 TYR907 HIS909 GLU988 ILE994
p53 (1TUP)	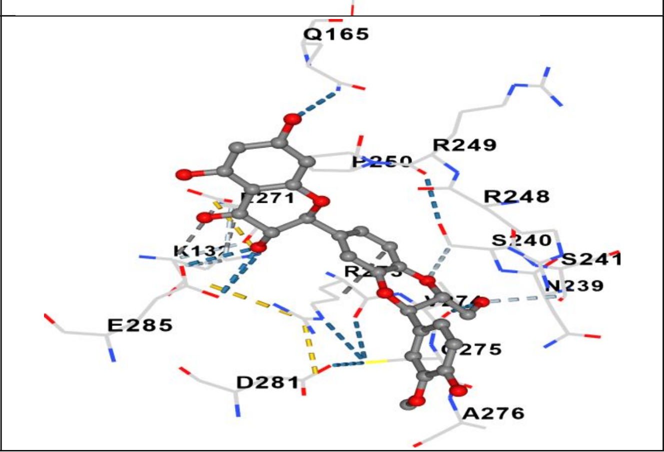	**Chain C**: LYS120 LEU130 LYS132 GLN136 LEU137 LYS139 LYS164 GLN165 ASN239 SER240 SER241 MET243 ARG248 ARG249 PRO250 ILE251 GLU271 VAL272 ARG273 VAL274 CYS275 ALA276 CYS277 ARG280 ASP281 THR284 GLU285 ASN288

<table>
<tr><td>CYCS (3ZCF)</td><td>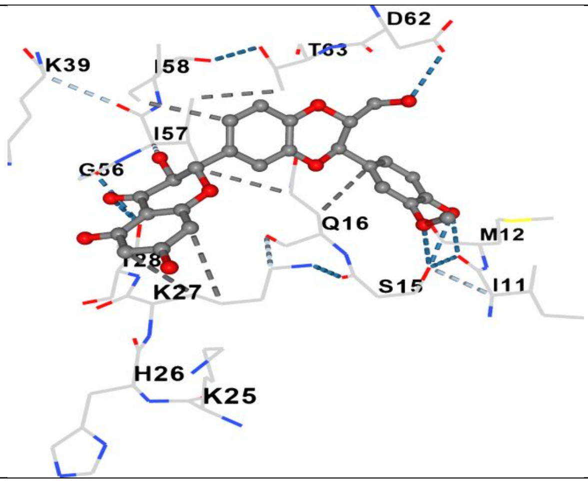
</td><td>Chain A: ILE11 MET12 LYS13 SER15 GLN16 CYS17 LYS25 HIS26 LYS27 THR28
Chain B: LYS39 GLY56 ILE57 ILE58 TRP59 GLY60 GLU61 ASP62 THR63 MET65 GLU66 GLU69 LYS88 ARG91</td></tr>
<tr><td>NRF2 (5WFV)</td><td>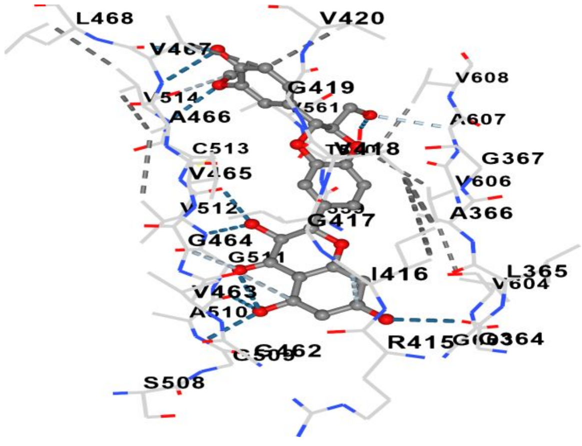
</td><td>Chain A: ARG326 GLY364 LEU365 ALA366 GLY367 CYS368 VAL369 VAL370 GLY371 ARG415 ILE416 GLY417 VAL418 GLY419 VAL420 GLY462 VAL463 GLY464 VAL465 ALA466 VAL467 LEU468 SER508 GLY509 ALA510 GLY511 VAL512 CYS513 VAL514 LEU515 HIS516 ALA556 LEU557 GLY558 ILE559 THR560 VAL561 HIS562 GLN563 GLY603 VAL604 GLY605 VAL606 ALA607 VAL608 THR609</td></tr>
<tr><td>KEAP1 (8X34)</td><td>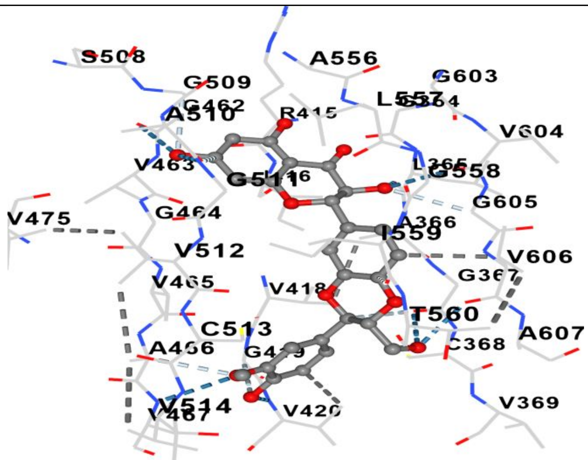
</td><td>Chain B: TYR334 SER363 GLY364 LEU365 ALA366 GLY367 CYS368 VAL369 ARG380 ASN382 ARG415 ILE416 GLY417 VAL418 GLY419 VAL420 ILE421 ILE461 GLY462 VAL463 GLY464 VAL465 ALA466 VAL467 VAL475 PHE478 ARG483 SER508 GLY509 ALA510 GLY511 VAL512 CYS513 VAL514 TYR525 GLN530 SER555 ALA556 LEU557 GLY558 ILE559 THR560 VAL561 TYR572 SER602 GLY603 VAL604 GLY605 VAL606 ALA607 VAL608</td></tr>
<tr><td>HO-1 (1N3U))</td><td>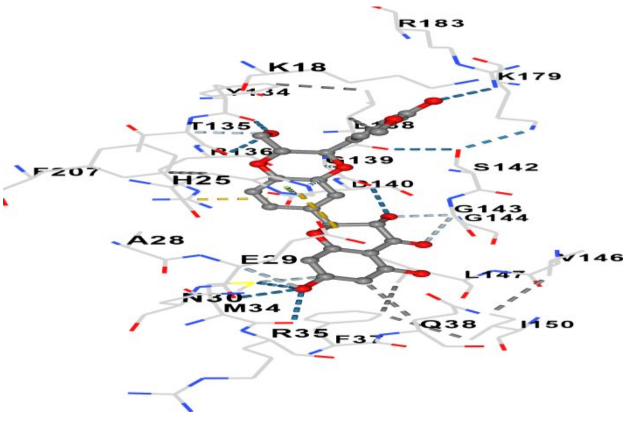
</td><td>Chain B: SER14 GLU15 LYS18 THR21 HIS25 ALA28 GLU29 ASN30 MET34 ARG35 PHE37 GLN38 VAL50 LEU54 TYR114 TYR134 THR135 ARG136 LEU138 GLY139 ASP140 SER142 GLY143 GLY144 VAL146 LEU147 ILE150 PHE166 PHE167 LYS179 ARG183 ALA206 PHE207 ASN210 PHE214</td></tr>
</table>

TNF-α (1TNF)	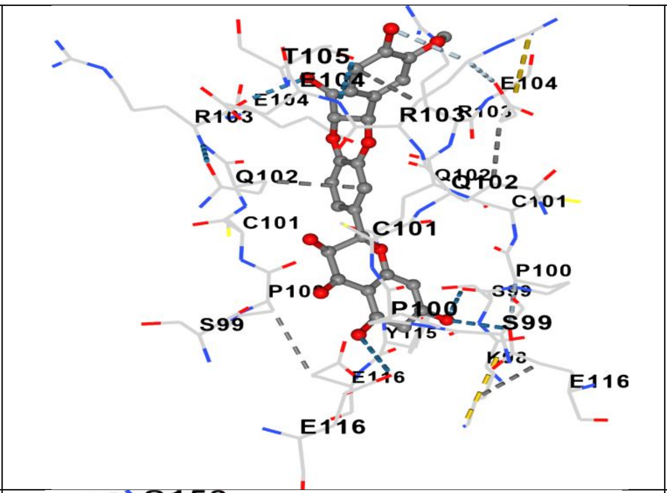	**Chain A**: LYS98 SER99 PRO100 CYS101 GLN102 ARG103 GLU104 THR105 PRO106 GLU107 GLY108 TRP114 TYR115 GLU116<br>**Chain B**: LYS98 SER99 PRO100 CYS101 GLN102 ARG103 GLU104 THR105 PRO106 GLU107 TRP114 TYR115 GLU116<br>**Chain C**: CYS69 LYS98 SER99 PRO100 CYS101 GLN102 ARG103 GLU104 TRP114 TYR115 GLU116
IL-1β (1I1B)	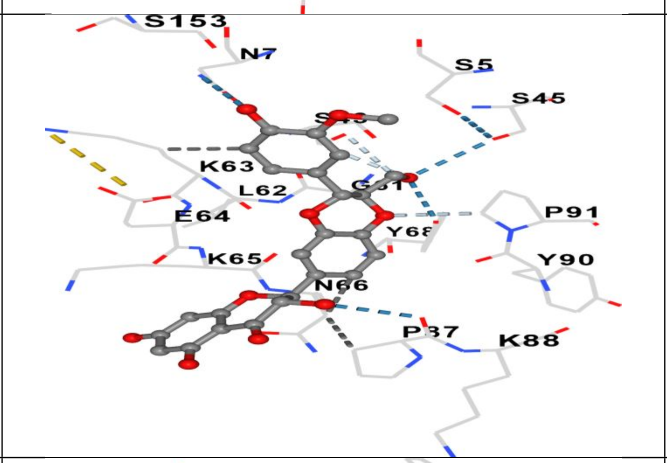	**Chain A**: VAL3 ARG4 SER5 LEU6 ASN7 SER43 SER45 GLY61 LEU62 LYS63 GLU64 LYS65 ASN66 LEU67 TYR68 VAL85 ASP86 PRO87 LYS88 TYR90 PRO91 VAL151 SER152 SER153
IL-6 (1ALU)	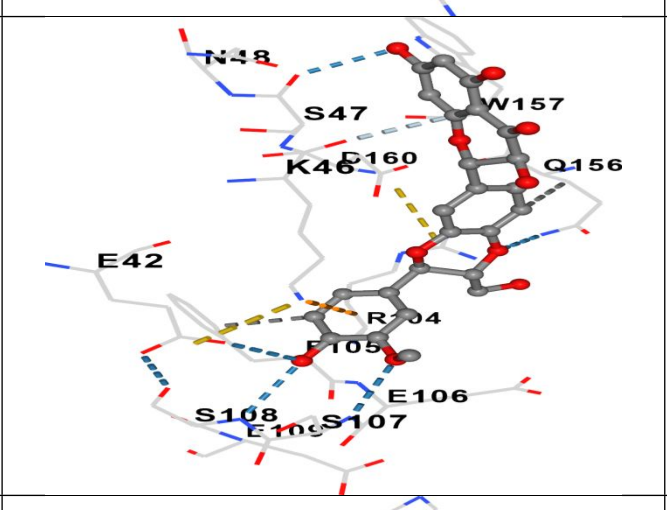	**Chain A**: GLU42 THR43 CYS44 LYS46 SER47 ASN48 MET49 ASN103 ARG104 PHE105 GLU106 SER107 SER108 GLU109 GLN152 ALA153 GLN154 GLN156 TRP157 GLN159 ASP160 THR163
NF-κβ p65 (4G3D)	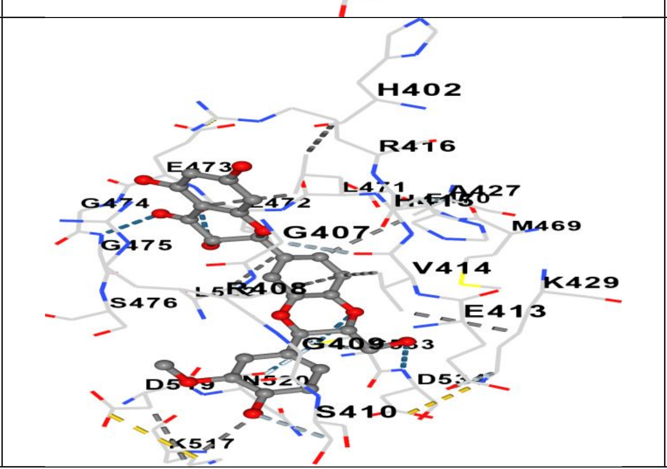	**Chain E**: GLU395 GLU396 THR401 HIS402 GLY407 ARG408 GLY409 SER410 PHE411 GLY412 GLU413 VAL414 HIS415 ARG416 ALA427 LYS429 LYS430 VAL431 ARG432 VAL435 TRP464 MET469 GLU470 LEU471 LEU472 GLU473 GLY474 GLY475 SER476 GLN479 LYS482 LYS517 ASP519 ASN520 LEU522 CYS533 ASP534

p38 MAPK (2Y8O)	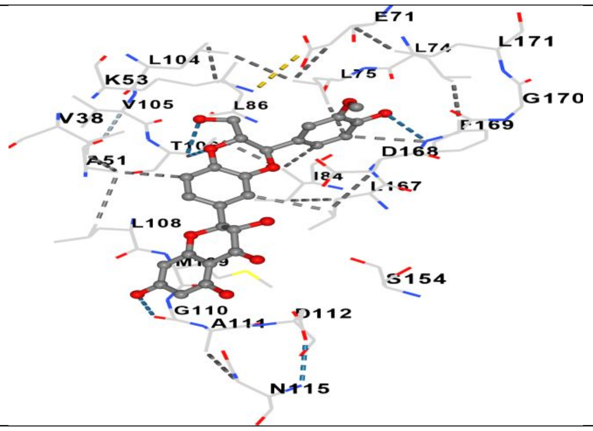	**Chain A**: VAL30 GLY31 SER32 GLY33 TYR35 VAL38 ALA40 ARG49 ALA51 LYS53 ARG67 GLU71 LEU74 LEU75 ILE84 LEU86 LEU104 VAL105 THR106 HIS107 LEU108 MET109 GLY110 ALA111 ASP112 ASN115 ASP150 LYS152 SER154 ASN155 LEU156 ALA157 VAL158 ILE166 LEU167 ASP168 PHE169 GLY170 LEU171 ALA172 THR185 ARG189 **Chain B**: PRO16
CASP9 (5JUY)	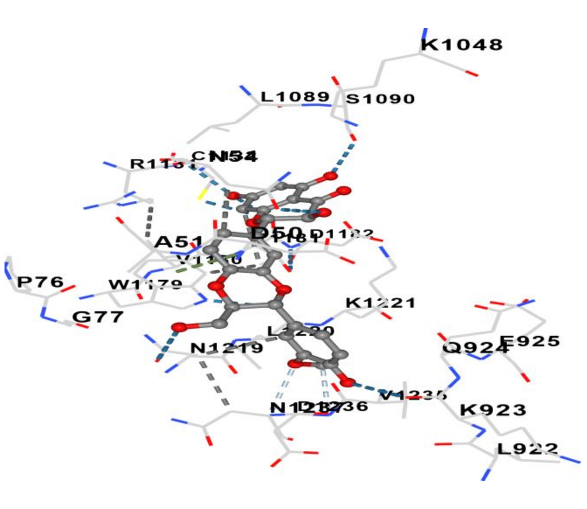	**Chain D**: HIS553 LEU554 ALA617 GLN861 TYR862 LEU882 SER883 TRP884 ASP902 GLN904 LEU922 LYS923 GLN924 GLU925 HIS1008 LYS1048 ASP1049 TRP1061 LEU1089 SER1090 ARG1131 CYS1132 ASN1148 GLY1149 GLU1150 PRO1167 LEU1168 SER1169 ALA1173 ALA1174 THR1175 HIS1176 GLY1177 GLY1178 TRP1179 VAL1180 THR1181 ASP1182 GLY1196 TYR1198 LYS1200 GLN1211 PHE1213 TYR1214 THR1218 ASN1219 LEU1220 LYS1221 LYS1222 VAL1235 ASP1236 ASN1237 LEU1238 GLY1239 **Chain K**: TYR48 THR49 ASP50 ALA51 ASN54 GLU69 ASN70 LYS72 LYS73 PRO76 GLY77 THR78 ILE81 ALA83
CASP8 (6PX9)	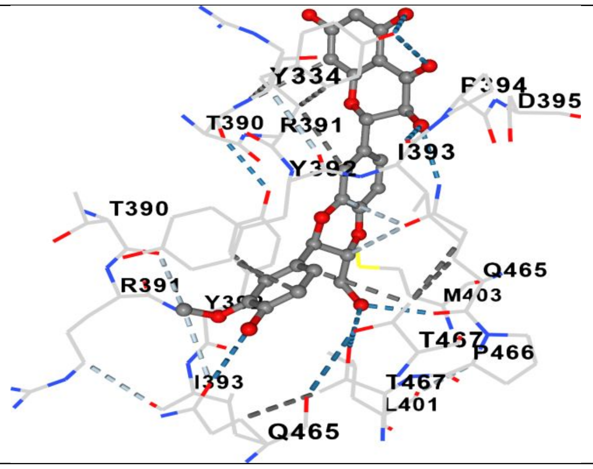	**Chain C**: ASP319 LYS320 GLY321 ILE322 PRO332 TYR334 GLU335 PRO388 GLN389 THR390 ARG391 TYR392 ILE393 LEU401 GLY402 MET403 ALA404 THR405 MET463 PRO464 GLN465 PRO466 THR467 **Chain D**: LYS320 TYR334 GLN361 THR390 ARG391 TYR392 ILE393 PRO394 ASP395 GLN465 THR467
AP-1 (4HMY)	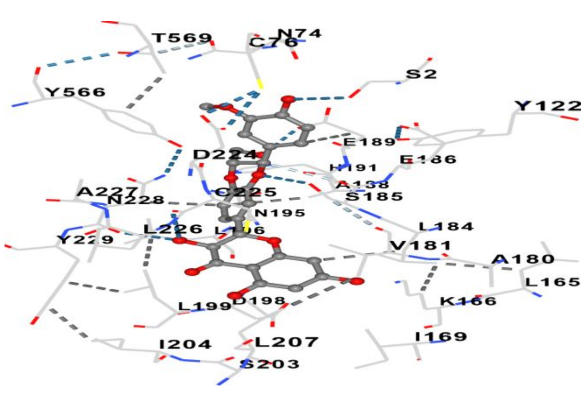	**Chain B**: LEU165 LYS166 ASP167 LEU168 ILE169 ALA180 VAL181 LEU184 SER185 GLU186 ALA188 GLU189 HIS191 ASN195 LEU196 LEU197 ASP198 LEU199 ASN200 SER203 ILE204 LEU207 LEU208 LEU211 ILE222 ASP224 CYS225 LEU226 ALA227 ASN228 TYR229 ILE240 TYR566 THR569 **Chain M**: SER2 ASN74 CYS76 TYR122
